# The Use and Reporting of the Cross-Over Study Design in Clinical Trials and Systematic Reviews: A Systematic Assessment

**DOI:** 10.1371/journal.pone.0159014

**Published:** 2016-07-13

**Authors:** Sarah Jane Nolan, Ian Hambleton, Kerry Dwan

**Affiliations:** 1 Department of Biostatistics, University of Liverpool, Liverpool, United Kingdom; 2 Cochrane Cystic Fibrosis and Genetic Disorders Group, Liverpool, United Kingdom; 3 Chronic Disease Research Centre, The University of the West Indies, Barbados, West Indies; 4 Cochrane Editorial Unit, London, United Kingdom; University Hospital Jena, GERMANY

## Abstract

**Background:**

Systematic reviews of treatment interventions in stable or chronic conditions often require the synthesis of clinical trials with a cross-over design. Previous work has indicated that methodology for analysing cross-over data is inadequate in trial reports and in systematic reviews assessing trials with this design.

**Objective:**

We assessed systematic review methodology for synthesising cross-over trials among Cochrane Cystic Fibrosis and Genetic Disorders Group reviews published to July 2015, and assessed the quality of reporting among the cross-over trials included in these reviews.

**Methodology:**

We performed data extraction of methodology and reporting in reviews, trials identified and trials included within reviews.

**Principal Findings:**

We reviewed a total of 142 Cochrane systematic reviews including 53 reviews which synthesised evidence from 218 cross-over trials. Thirty-three (63%) Cochrane reviews described a clear and appropriate method for the inclusion of cross-over data, and of these 19 (56%) used the same method to analyse results. 145 cross-over trials were described narratively or treated as parallel trials in reviews but in 30 (21%) of these trials data existed in the trial reports to account for the cross-over design. At the trial level, the analysis and presentation of results were often inappropriate or unclear, with only 69 (32%) trials presenting results that could be included in meta-analysis.

**Conclusions:**

Despite development of accessible, technical guidance and training for Cochrane systematic reviewers, statistical analysis and reporting of cross-over data is inadequate at both the systematic review and the trial level. Plain language and practical guidance for the inclusion of cross-over data in meta-analysis would benefit systematic reviewers, who come from a wide range of health specialties. Minimum reporting standards for cross-over trials are needed.

## Introduction

A cross-over trial is a design in which participants receive two or more sequential interventions in a random order in separate treatment periods, often separated by a washout period to avoid a ‘carry-over’ intervention effect from one treatment period into the next [1]. Such a design has advantages over a parallel design in which participants are allocated to a single intervention for comparison with other interventions. In a cross-over trial, each participant can act as their own control in the trial [2, 3], reducing the sample size required for the same statistical power, which is advantageous for assessing interventions for rare diseases. Cross-over designs are suitable for evaluating interventions with a temporary effect on stable or chronic conditions, such as arthritis, asthma or epilepsy [4]. Therefore, systematic reviews in these clinical areas often require the synthesis of intervention trials with a cross-over design.

Elbourne *et al* [5] reported that systematic review descriptions of cross-over trial synthesis were insufficient, and recommendations were made for improvement. Based on these recommendations, the Cochrane Handbook for Systematic Reviews of Interventions [6] has proposed a “three stage” decision process for including cross-over data in meta-analysis.

Ideally (first stage), the results from paired analyses, which adjust for within-individual comparisons, should be used. If the first stage approach is not possible, the second stage would be to include data from the first cross-over period, treating this period as a randomised parallel trial. Such an approach that requires trials to report data by treatment period and would result in a loss of statistical power from analysing only half of the available information from the trial. The third stage, the least desirable and most conservative approach, would be to assume the treatment arms are independent, which ignores the cross-over design and will likely overestimate variability of the within-study treatment effect. Despite the development of the recommendations in Elbourne *et al* [5], and subsequent guidance on meta-analyses combining parallel and cross-over data [7–9], it remains unclear whether these recommendations have been widely adopted in systematic reviews and meta-analyses, or how commonly the use of such ideal approaches is feasible from information reported in publications of cross-over trials.

For an appropriate trial synthesis, a systematic review needs access to methodological details of a trial and either individual level data or appropriately summarised data. Recent work found that cross-over trial reports often omit important methodological issues in design, presentation and analysis [4]. While Consolidated Standards of Reporting Trials (CONSORT) guidelines for randomised controlled trials in general have existed for nearly 20 years [10, 11], as yet the CONSORT reporting guidelines have not been extended specifically for cross-over trials.

Our objectives in this study were to assess review methodology for including cross-over trials in reviews published by the Cochrane Cystic Fibrosis and Genetic Disorders (CFGD) Group based on the “three stage” approach as described in Elbourne *et al* [5]. Our aims were to assess the quality of reporting of cross-over trials within the trial reports themselves and subsequently, within the Cochrane CFGD reviews and to establish the basis of practical guidelines for how cross-over data should be included in systematic reviews, in line with Methodological Expectations of Cochrane Intervention Reviews (MECIR) standards.

## Methods

### Selection of reviews and cross-over trials

We accessed the most recently published version of all Cochrane Cystic Fibrosis and Genetic Disorders (CFGD) reviews (published to July 2015) and recorded the number and designs of included studies in each systematic review. We excluded reviews that explicitly considered the cross-over trial design to be inappropriate given the review question, usually because an intervention effect was not considered to be temporary, making a washout period ineffectual. For 17 reviews it was unclear whether cross-over studies would be included, and we contacted the corresponding author of these reviews for clarification.

For all included systematic reviews, we recorded how review authors planned to synthesise cross-over trial data. If a review included at least one cross-over trial, we compared the actual synthesis method to the intended method.

We accessed reports of all included cross-over trials and recorded trial characteristics, analysis details and presentation of all trial results. For review primary outcomes, we compared the relevant trial level results to the data included in the review(s).

### Data extraction and presentation of results

Information extracted from included systematic reviews and cross-over trials are described in S1 Table, with data items extracted from cross-over trials based on a previous review of cross-over trial quality [4].

Two authors (SJN and KD) extracted information from reviews and trials using pre-designed data extraction forms. The third author (IH) independently double data extracted from a random sample of trials with uncertainties or discrepancies resolved by discussion.

Results of assessments at review and trial level are summarised narratively. Frequency data are presented as numbers and percentages.

## Results

### Selection of reviews and cross-over trials

**Fig 1:**
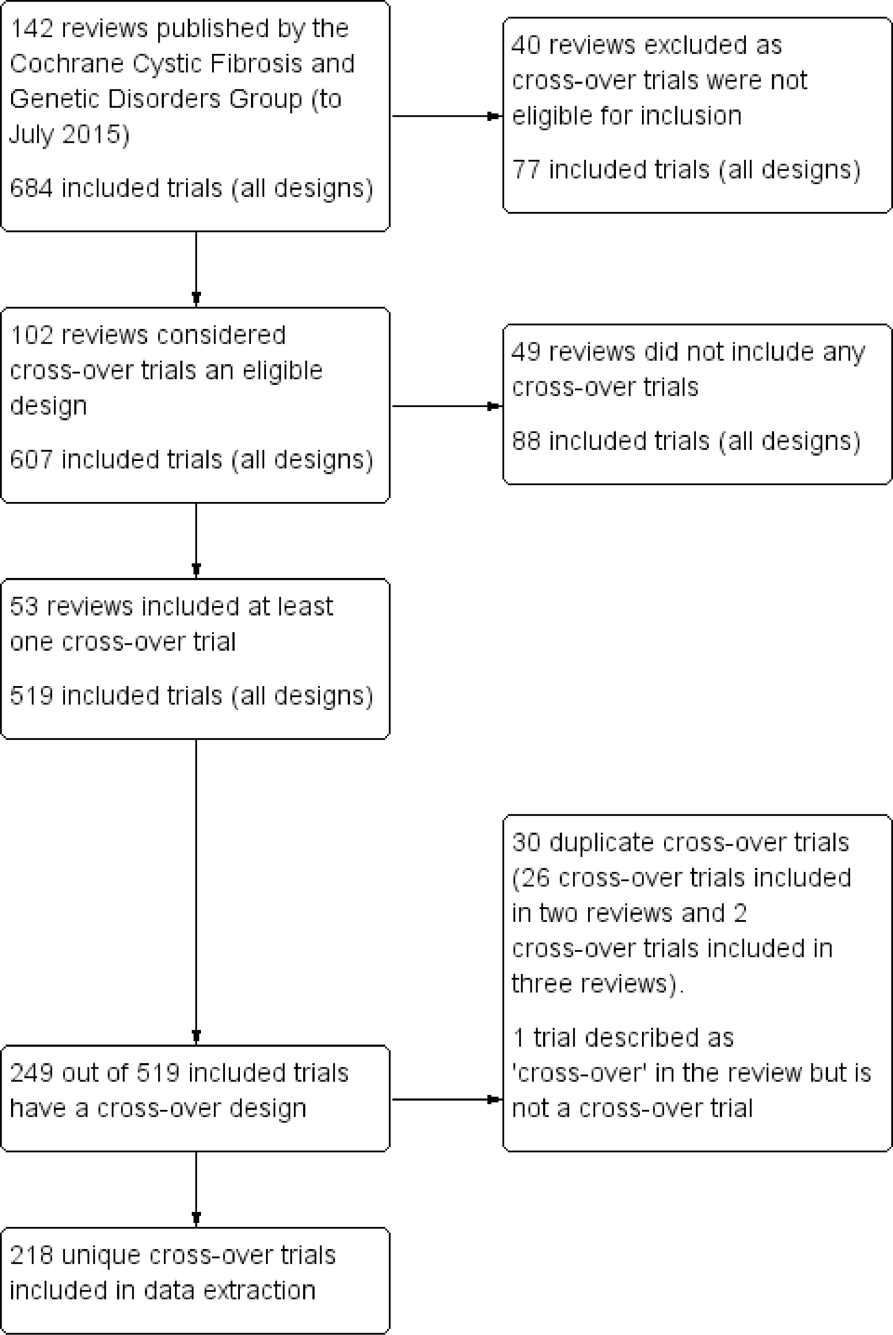
Study Flow Diagram. Flow diagram of selection of Cochrane Cystic Fibrosis and Genetic Disorders (CFGD) reviews and included cross-over trials^a-d^. ^a^ All numbers in Fig 1 refer to number of trials, some of which were published across multiple reports. Where this was the case, we extracted only from the primary reference as stated in the Cochrane review or according to our judgement of which reference was the most relevant. ^b^ In one review, review level and included trial level data was extracted following initial identification of reviews and trials up to January 2015. An update of this review was published in May 2015 in which five cross-over trials previously included were excluded (for reasons not due to cross-over design). These excluded cross-over trials were retained in the data extraction and results. ^c^ Note: in forty reviews which did not consider cross-over trials to be an eligible design; 13 cross-over trials were listed as “Excluded Studies” in these reviews due to design (one of which was included in another review). ^d^ Note: in the 102 reviews where cross-over designs were eligible, 10 unique trials listed as ‘Ongoing’ had a cross-over design and 35 unique trials listed as ‘Awaiting Assessment’ had a cross-over design (three of which were included in other reviews).

See Fig 1 for flow diagram of selection of reviews and cross-over trials, S1 File for reference list of included Cochrane CFGD reviews and S2 File for reference list of included cross-over trials.

Up to July 2015, 142 published CFGD reviews included a total of 684 trials of different designs. Cross-over designs were considered eligible in 102 reviews, and these reviews included 607 trials of any design. At least one cross-over trial was included in 53 reviews; twenty reviews included one cross-over trial, 11 reviews included two cross-over trials, 14 reviews included 3 to 10 cross-over trials and 8 reviews included 11 to 20 cross-over trials. A total of 218 unique cross-over trials; published between 1966 and 2012, were included in one or more of these 53 reviews. Less than 50% of the trials included were cross-over trials in 22 reviews, 50 to 99% of the included trials were cross-over trials in 21 reviews and in 10 reviews, all included trials were cross-over trials.

### Systematic review methods

**Fig 2:**
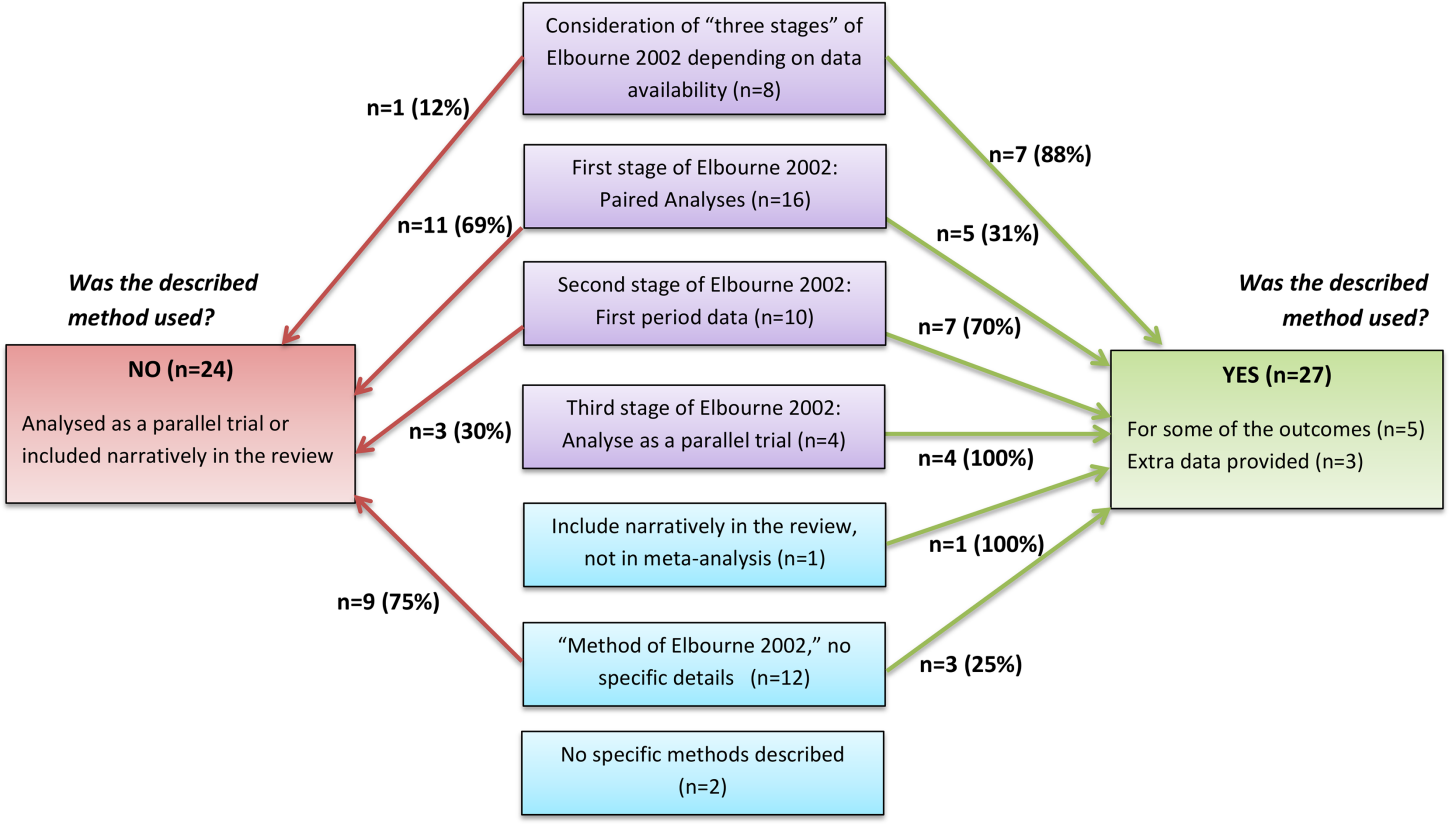
Review Methodology and Reporting. Methods planned compared to methods used for and the inclusion of results from cross-over (CO) trials in meta-analysis for 53 reviews.

Table 1 and Fig 2 summarise the methods planned and the methods used in CFGD reviews for the inclusion of cross-over data.

**Table 1:** Review methodology and reporting.

Methods described for the inclusion of cross-over (CO) trials in reviews	CO trials eligible (n = 102)	CO trials included (n = 53)
Describes “Three stages of Elbourne” approach [5]^a^	17 (17%)	8 (15%)
Use paired analyses (First stage of Elbourne)	16 (16%)	11 (21%)
Use generic inverse variance (GIV) meta-analysis (paired analysis)	6 (6%)	4 (7%)
Marginal probabilities of success method [12] (paired analysis)	3 (3%)	1 (2%)
Include first period data only (Second stage of Elbourne)	18 (17%)	10 (19%)
Analyse as a parallel trial (Third stage of Elbourne)	4 (4%)	4 (7%)
Refers to "Elbourne," no specific details of methods.	20 (19%)	12 (23%)
Include narratively in the review only	1 (1%)	1 (2%)
Consult a statistician	1 (1%)	1 (2%)
No methods stated	14 (14%)	1 (2%)
Consult the Cochrane Handbook	1 (1%)	0 (0%)
“Depends on study”	1 (1%)	0 (0%)
**Methods actually used in the reviews (n = 53 reviews with CO trials included)**		
***Same approach for all CO trials (one or more CO trials included in the review)*:**		
Included narratively in the review		15 (28%)
Analysed as a parallel trial		8 (15%)
Included first period data only		6 (11%)
Paired analyses (analysed by GIV meta-analysis)		5 (9%)
Paired analyses (data provided from trialist)		2 (4%)
First period (data provided from trialist)		1 (2%)
***Different approaches by CO trial (more than one CO trial included in the review)*:**		
Included narratively / analysed as a parallel trial		9 (17%)
First period only / analysed as parallel / Included narratively		5 (9%)
Paired analyses / first period only / analysed as parallel / Included narratively		2 (4%)
**Were the planned methods actually used? (n = 53 reviews with CO trials included)**		
NA—No specific analysis method described		2 (4%)
No—A method of paired analysis was described but data was analysed as parallel or data was included narratively		24 (45%)
Yes—for some of the studies / outcomes included in the review		5 (9%)
Yes—but were provided with extra data from trial authors		3 (6%)
Yes—specified three stages of Elbourne [5]^a^ and analysed data as parallel (third stage)		6 (11%)
Yes—method of including CO trial data used as described		13 (25%)

Legend: Methods planned compared to methods used for and the inclusion of results from cross-over (CO) trials in meta-analysis.

^a^ See Introduction for further details.

No methods or vague methods such as “consult a statistician” were described in 17 out of the 102 reviews (17%); but only two of these reviews actually included at least one cross-over trial. Fifty-three of the 102 reviews cite Elbourne *et al* [5], with 20 out of these 53 reviews (37%) giving no further description of their intended methods. Only a single review planned to include cross-over results narratively in the review only. The remaining 64 reviews described one or more of the “three stages” from Elbourne *et al* [5] for selecting and analysis approach, with 18 reviews intending to include first period data only and 4 reviews intending to conservatively analyse cross-over trials as parallel trials.

In the 53 reviews which included cross-over trials, 16 reviews with more than one cross-over trial included used more than one approach to analysing the trials (Table 1). Twenty seven out of the 53 reviews used the method described in the ‘Methods’ section of the review; three were provided with extra data by the original trialists. The other 24 reviews which described a method of paired analyses or analysis of first period data actually included studies narratively or analysed as parallel studies (Fig 2); it was mostly unclear whether authors had chosen not to use the analysis method described or if it was not feasible to use the method due to presentation of results.

### Cross-over trial reporting

**Fig 3:**
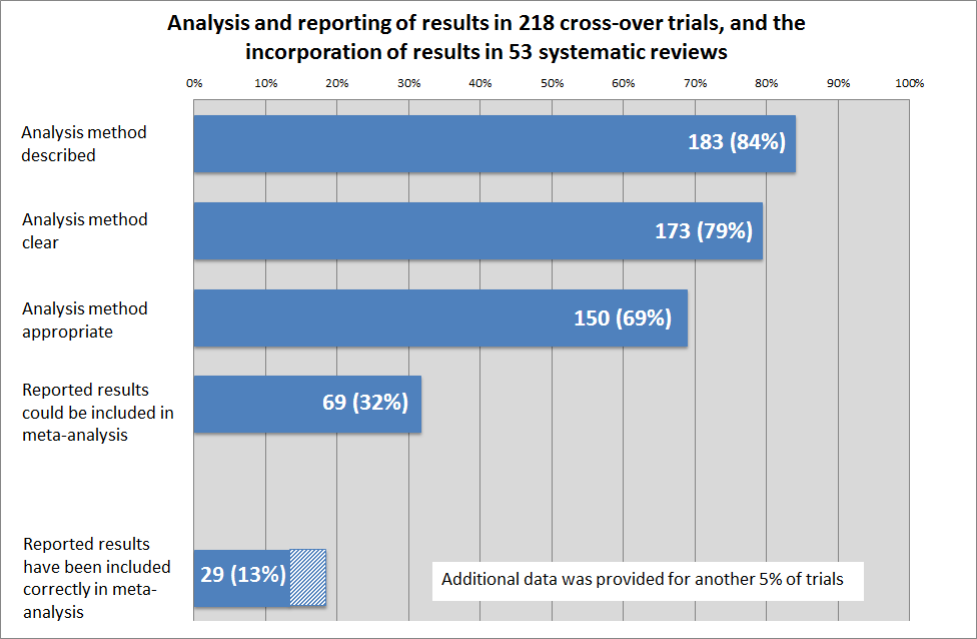
Trial methodology and reporting. Analysis and reporting of results in 218 cross-over trials, and the incorporation of results in 53 systematic reviews.

**Fig 4:**
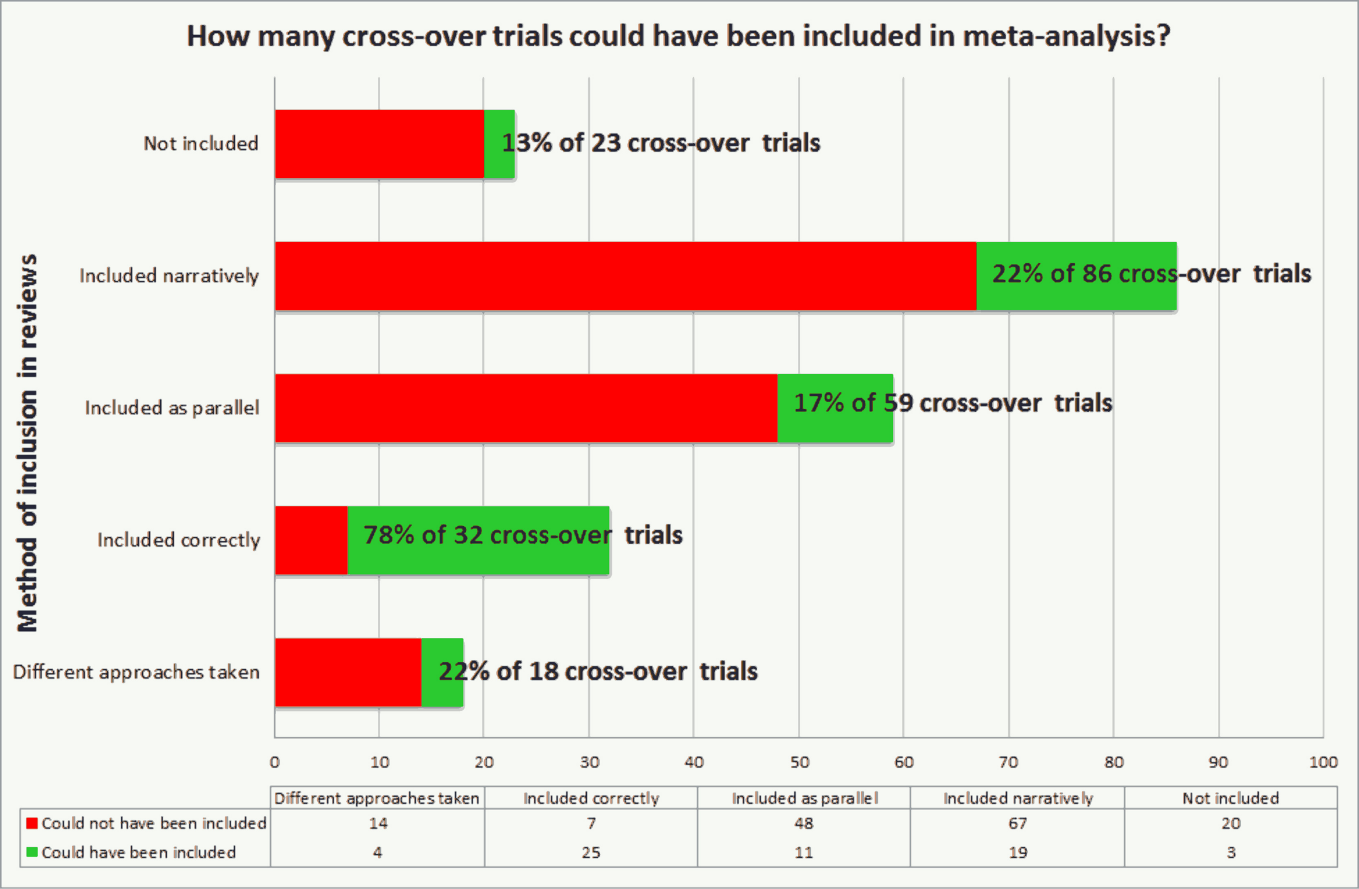
Inclusion of cross-over data in systematic reviews. How many cross-over trials could be been included in meta-analysis?

Table 2, Fig 3 and Fig 4 summarise the characteristics, analysis details and presentation of the results in the 218 cross-over studies.

**Table 2:** Characteristics, statistical analysis and presentation of results in cross-over trials.

Trial design and characteristics (n = 218)	
***Cross-over design*^*a*^^,^^*b*^**	
AB/ BA design (i.e. randomised to one of two interventions and then order reversed)	157 (72%)
More than two arms in a randomised order	44 (20%)
Other design	14 (7%)
Unclear	3 (1%)
***Washout period*** ^***c***^	
Used	60 (27%)
Not used	28 (13%)
Not mentioned	119 (55%)
Unclear	11 (5%)
***Clinical justification of cross-over design*?**	
Not mentioned	203 (93%)
Control within-participant variability (participant acts as own control)	6 (3%)
States the measurement of participant preference is an objective	6 (3%)
Increase the statistical power of a small sample size (rare condition)	3 (1%)
***Participant preference measured as an outcome*?**	
Yes	42 (19%)
No	176 (81%)
***Sample size calculation takes account of cross-over design*?**	
No calculation specified	177 (81%)
The study is a pilot or exploratory so a sample size calculation is not necessary	6 (3%)
Sample size calculation described but no allowance for cross-over design	27 (12%)
Sample size calculation allows for paired / cross-over design	8 (4%)
**Statistical analysis (n = 218)**	
***Carry-over effect***	
Not mentioned	169 (77%)
Text regarding carry-over effect but no statistical test performed:	21 (10%)
*Baseline comparisons made across treatment periods*	*7 (33%)*
*Washout period removes cross-over effects*	*6 (29%)*
*Short treatment effect so no carry-over*	*1 (5%)*
*"No carry-over effect"*	*5 (24%)*
*Exclusion of first month of each period / trial starts with a run in period*	*2 (9%)*
Carry-over effect described as "significant" or "non-significant effect" but not stated which statistical test was used	9 (4%)
Statistical test for carry-over specified:	19 (9%)
*ANOVA / ANCOVA*	*9 (48%)*
*Method of Hills & Armitage [13]*	*3 (16%)*
*Mixed effects model*	*4 (21%)*
*Wilcoxon Signed Rank test*	*1 (5%)*
*Mann Whitney test / Wilcoxon Rank Sum test*	*1 (5%)*
*Paired t-test*	*1 (5%)*
***Period effect***	
Not mentioned	170 (78%)
Text regarding period effect but no statistical test performed:	9 (4%)
*The randomised order avoids period effect*	*3 (34%)*
*Baseline comparisons made across treatment periods*	*2 (22%)*
*"No period effect"*	*2 (22%)*
*Possible seasonal / order variations (separate presentation of seasonal results)*	*2 (22%)*
Period effect described as "significant" or "non-significant effect" but not stated which statistical test was used:	12 (6%)
*Analysis adjusted for period effect*	*3 (25%)*
*Analysis did not adjust for period effect*	*2 (16%)*
*Not stated whether period effect was adjusted for in analysis*	*7 (58%)*
Statistical test for period effect specified:	27 (12%)
*ANOVA / ANCOVA*	*11 (41%)*
*Method of Hills & Armitage [13]*	*2 (7%)*
*Mixed effects model*	*6 (23%)*
*Wilcoxon Signed Rank test*	*4 (15%)*
*Mann Whitney test / Wilcoxon Rank Sum test*	*2 (7%)*
*Paired t-test*	*2 (7%)*
*Analysis adjusted if period effect was present (for trials with a statistical test for period effect)*:	27 (12%)
*Analysis adjusted for period effect (period effect present)*	*5 (19%)*
*Analysis did not adjust for period effect (period effect not present)*	*2 (7%)*
*Not stated whether period effect was present or adjusted for in analysis*	*20 (74%)*
***Appropriate statistical analysis performed*?**^***d***^	
No statistical methods described	35 (16%)
Statistical methods not clear:^e^	10 (4%)
*Described as "Non-parametric methods" or "Regression*,*" no further details*	*5 (50%)*
*“Paired t-test or Wilcoxon Rank Sum test” or "Paired and unpaired t-tests"*	*4 (40%)*
*Study "analysed as cross-over*,*" no further details*	*1 (10%)*
Statistical analysis not appropriate:	23 (11%)
*ANOVA (not repeated measures)*	*11 (48%)*
*T-test (unpaired)*	*5 (22%)*
*Mann Whitney test / Wilcoxon Rank Sum Test (unpaired)*	*5 (22%)*
*Binomial test*	*2 (8%)*
Analysis appropriate for paired design:	150 (69%)
*Paired t-test*	*52 (35%)*
*Wilcoxon Signed Rank tests*	*31 (20%)*
*Combination of paired t-test / Wilcoxon Signed Rank / Repeated Measures ANOVA / Friedman's ANOVA [14]* ^*g*^	*27 (18%)*
*Repeated Measures ANOVA or ANCOVA*	*24 (16%)*
*Methods of Hills & Armitage [13]*	*5 (3%)*
*Mixed effects models*	*4 (3%)*
*Analysis of first period data only*	*3 (2%)*
*McNemar’s test*	*2 (1%)*
*Methods described by Grizzle [15]*	*1 (1%)*
*Methods described by Lehmacher [16]*	*1 (1%)*
***Intention to treat approach used to analysis*?**	
Yes	69 (32%)
No	44 (20%)
Unclear if any participants have been excluded from analysis	105 (48%)
***Study flow diagram of participants presented*?**	
Yes	13 (6%)
No	205 (94%)
**Presentation of results (n = 218)** ^*g*^	
Published results could be included in meta-analysis	56 (26%)
*Adjusted results presented (or enough information to calculate the adjusted results)*	*21 (37%)*
*Individual participant data presented (tabular or graphical)*	*24 (43%)*
*Results presented by treatment period*	*6 (11%)*
*First period results only presented*	*5 (9%)*
Results for some outcomes could be included in meta-analysis	13 (6%)
*Some results presented as parallel*, *some individual participant data presented*	*8 (62%)*
*Some results presented as parallel*, *some adjusted results presented*	*2 (15%)*
*Some results presented as parallel*, *some results presented by treatment period*	*3 (23%)*
Published results could not be included in meta-analysis accounting for cross-over design.	149 (68%)
*Results presented as parallel (i*.*e by treatment group*, *end of the study*, *non-exact p values)*	*122 (82%)*
*Narrative description of results only (no summary statistics presented)*	*14 (9%)*
*Some individual participant data reported but not enough to use*	*11 (7%)*
*Results reported according to participant subgroups rather than by treatment*	*2 (1%)*

ANOVA: Analysis of variance, ANCOVA: Analysis of covariance

^a^ Examples of other designs: participants received at least one of the interventions more than once (e.g. two treatment periods of each intervention).

^b^ Examples of unclear designs: unclear how interventions were allocated or when cross-over occurred.

^c^ Unclear washout period: for example, a gap between treatments is described (e.g. interventions were given on consecutive days) but unclear if this was intended to be a washout period.

^d^ Statistical analyses considered appropriate *a priori* are described in S1 Table. Other methods considered on a case by case basis

^e^ Statistical analysis section describes as mixture of paired and unpaired tests; e.g paired t-test if data is normally distributed and Wilcoxon Rank Sum test (not paired) if data is skewed.

^f^ Non-parametric equivalent of repeated measures ANOVA.

^g^ Data could be included in meta-analysis if results adjusted for the paired design could be extracted (e.g. mean difference and standard error (SE) of mean difference) or calculated (e.g. from individual participant data presented, from estimation of SE from exact p value reported or from correlation coefficient between treatment groups) or if data was presented by treatment period or first period only.

The trials were of mostly small sample size ranging from 4 to 116 participants (median sample size 18). The majority of trials (72%) had an AB/BA design (i.e. participants randomised to one of two interventions and then order reversed). The use of a washout period was clearly described in 60 trials (27.5%), and assessment for a carry-over or period effect was clearly described in 48 trials (22%; Table 2).

Forty-five trials (20%) described no or unclear statistical methods and in a further 23 trials (11%), an inappropriate method of statistical analysis was described for the cross-over design (i.e. a statistical test for independent groups). The other 150 trials (69%) described an appropriate method of statistical analysis for the paired design (Fig 3).

In 69 (32%) trials, sufficient information was presented to include some or all results in meta-analysis; either adjusted results, individual participant data or results according to treatment period (Fig 3). In the remaining 149 (68%) trials, the presentation of the results would not allow the inclusion in meta-analysis; most trials presented results narratively or as a parallel trial.

### How cross-over trials were included in systematic reviews

S2 Table, Fig 3 and Fig 4 summarise reporting of cross-over trial data in publications and how published results were included in the CFGD reviews.

One hundred and ninety trials were included in a single review, 26 trials were included in two reviews and two trials were included in three reviews; a full description of how trials were included in meta-analysis is given in S2 Table.

Twenty-three trials (11%) were not included in the results of the reviews due to no outcomes of interest to the review reported or ongoing information requests to original trial authors. A further 86 trials (39%) were included narratively in the review but not in meta-analysis; 19 of which could have been included in meta-analysis from published information provided (Fig 4).

Thirty-two trials (15%) were included correctly accounting for design in meta-analysis via the analysis of adjusted results or analysis of first period data (Fig 4). In seven of these trials, sufficient information was not presented in the trial reports; it was stated for three trials that extra data was provided by trialists but for the other four trials, results seemed to have been adjusted for inclusion in the review but it was not stated that extra information was provided.

Fifty-nine trials (27%) were included in reviews as parallel trials; 11 of which could have been included correctly accounting for design in meta-analysis (Fig 4). The remaining 18 trials (8%) were included in two or more reviews and different approaches to the inclusion of the same results were taken across the reviews (S2 Table).

## Discussion

### Statement of principal findings

The cross-over trial design is commonly used in chronic, stable, and rare disorders. Out of the 684 unique trials included in 142 reviews published by the Cochrane Cystic Fibrosis and Genetic Disorders (CFGD) Group up to July 2015, around a third (218 unique trials) had a cross-over design.

Despite the wide use of such designs in this area, only around 60% of CFGD reviews describe a clear and appropriate method for the inclusion of cross-over data in the review and only around half of these reviews eventually used their proposed synthesis methodology.

The analysis and presentation of results in cross-over trial reports were often inappropriate or unclear. Around 70% of trials clearly described appropriate statistical methodology but only 30% presented results that could be included in meta-analysis. Nearly 80% of studies failed to acknowledge or assess the presence of carry-over or period effect; biases which if present must be accounted for when interpreting results and performing future meta-analyses

Cross-over trials were most commonly included narratively in CFGD reviews, rather than in meta-analysis; however 30% of cross-over trials were included in meta-analysis incorrectly for design as parallel trials. In addition to ignoring carryover and period effects, the conservative assumption of parallel treatment arms over-estimates the variability between the treatment groups, artificially widening confidence intervals of the pooled treatment effect in meta-analysis. For the 28 cross-over trials which were included in more than one review, different approaches were taken for 18 of these trials (64%) by review authors to including the same results.

### Strengths and limitations of the study

The strength of this study is the systematic, detailed assessment of all published reviews of a Cochrane Review Group and all cross-over trials within them.

We took the approach of assessing the most recently published version of reviews and made the assumption that methods described reflected an *a priori* analysis plan of cross-over data. However, it is possible that originally planned methods had been updated over time as Cochrane reviews were updated or adapted to reflect the approach actually taken in the review or in accordance with changes in Cochrane reporting standards over time. To know originally planned methods an assessment of original review protocols would be required. Such a protocol assessment had its own drawbacks, with some of the included reviews initiated up to 20 years ago, before the initiation of current format and current guidelines for Cochrane review protocols. Therefore this study does not provide an assessment of a change in reporting standards over time, but does highlight some reporting inconsistencies between methods and results sections of the most up to date version of each review.

We also note that the scientific aims of a review may influence the proposed methodology; for example, analysis of first period data may be preferable to analysis of paired results if carry-over is a particular concern for review authors. It was out of the scope of this work to examine clinical objectives of reviews in detail; however we encourage systematic reviewers to clearly state and justify use of particular methodology to address review specific clinical objectives.

When reviewing the inclusion of cross-over trials in systematic reviews, we only considered primary endpoints of Cochrane reviews. It would be inadvisable for Cochrane systematic reviews to vary synthesis methodology according to whether an endpoint is considered as primary or secondary. Therefore it is likely that if secondary review outcomes had also been considered, results would be similar.

### Comparison to other studies

The poor reporting of cross-over trials in this assessment broadly agrees with the work of Mills *et al* [4], which considered all cross-over trials published within an 18 month period and concluded that cross-over trials “frequently omit important methodological issues in design, analysis, and presentation. “The sample of cross-over trials included in this current assessment is from specific disease areas, in contrast to Mills’ wide inclusion [4]. Nevertheless, our results highlight similarly poor reporting and in some areas, such as the use of inappropriate or unclear statistical methods and lack of clarity regarding use of washout period, are worse.

Elbourne *et al* [5] concluded that “poor reporting of cross-over trials will often impede attempts to perform a meta-analysis using the available methods.” This study shows that the reporting of cross-over trials still impedes their inclusion in meta-analysis, with inadequate presentation of results preventing data inclusion in meta-analysis for nearly 70% of cross-over trials assessed.

The methods of Elbourne *et al* [5] were cited in over half of the CFGD reviews which considered cross-over trials to be eligible, indicating some level of awareness of methodology for meta-analysis involving cross-over trials. However, it was not demonstrated in a large proportion of the reviews citing Elbourne *et al* [5] that the authors understood the methodology or how to implement the described approaches.

The results of this study are also largely comparable to the review of Lathrytis *et al* [17]; evaluating the analysis and results of cross-over trial data in a sample of Cochrane review meta-analyses compared to parallel design trials. The authors report variability in the approach to analysis of cross-over data and a paucity of clear methodological information regarding analysis approach. Our results suggest that despite nearly ten years of development of accessible guidance and training for Cochrane systematic reviewers, reporting of methodology related to cross-over data has not improved.

### Recommendations and future research

This study has important implications for future research at both the level of systematic reviews and trial level. Current guidance available to systematic review authors on the meta-analysis of cross-over data is often statistically technical and published in specialised journals [5, 7–9] and even in the Cochrane Handbook for Systematic Reviews of Interventions [6], analysis of cross-over data is covered under the chapter of “Special Topics in Statistics” [18]. The inconsistency and lack of detail in described methodology, and the variability in approaches to the inclusion of results in CFGD reviews suggests that review authors require more practical, plain language guidance for the inclusion of cross-over data in systematic reviews, to supplement the current statistical based guidance.

We note that while cross-over designs are appropriate for many systematic review questions within the scope of the CFGD review group, the proportion of cross-over trials included, and the review author guidance for the inclusion of cross-over data may vary by clinical area and by Cochrane Review Group. Therefore, it is important that guidance given to systematic review authors should be consistent when given at a review group level, but appropriate for the scope of the review group. An extension of this study could consider the review methodology for the inclusion of cross-over data and the quality of reporting of included cross-over trials of other Cochrane review groups.

Mills *et al* [4] called for development of “minimum standards for the transparent reporting of cross-over trials.” The present study emphasises the need for the development of reporting standards such as an extension of the CONSORT guidelines, for cross-over studies. The existence of minimum reporting standards at a trial level, in addition to supplementary guidance for systematic reviewers analysing cross-over data could increase the proportion of meta-analyses adequately conducted; which would be greatly beneficial to clinical decision making, particularly in clinical areas where cross-over designs are commonly used.

## Conclusion

Statistical analysis and reporting of cross-over data is inadequate at both the systematic review and the individual trial level. Plain language and practical guidance for the inclusion of cross-over data in Cochrane meta-analyses at a review group level are needed. Minimum reporting guidelines, such as a CONSORT extension to cross-over trials are needed to ensure that results from trials of such design can be appropriately synthesised.

## Supporting Information

S1 File Cochrane Cystic Fibrosis and Genetic Disorders Group reviews. Reference list of 142 Cochrane Cystic Fibrosis and Genetic Disorders reviews published to July 2015.
(DOCX)

S2 File Cross-over trials included in Cochrane Cystic Fibrosis and Genetic Disorders Group reviews. Reference list of 218 unique cross-over trials included in Cochrane Cystic Fibrosis and Genetic Disorders reviews published to July 2015.
(DOCX)

S1 Table Data items extracted. List of information extracted at the review level and at the study level.
(DOCX)

S2 Table Inclusion of cross-over data in Cochrane Reviews. Inclusion of cross-over trials for the primary outcomes (PO) of reviews of the Cochrane Cystic Fibrosis and Genetic Disorders Group.
(DOCX)
