# Supplementary material for: The Use and Reporting of the Cross-Over Study Design in Clinical Trials and Systematic Reviews: A Systematic Assessment
Source: PLoS One. 2016 Jul 13;11(7):e0159014. doi: 10.1371/journal.pone.0159014 (PMC4943623; doi:10.1371/journal.pone.0159014)
Supplement: S1 File — Reference list of 142 Cochrane Cystic Fibrosis and Genetic Disorders reviews published to July 2015. (DOCX) [file pone.0159014.s001.docx]

**Supplementary File 1: Cochrane Cystic Fibrosis and Genetic Disorders Group reviews.** Reference list of 142 Cochrane Cystic Fibrosis and Genetic Disorders reviews published to July 2015.

Active cycle of breathing technique for cystic fibrosis

Angiotensin-converting enzyme (ACE) inhibitors for proteinuria and microalbuminuria in people with sickle cell disease

Antibiotic adjuvant therapy for pulmonary infection in cystic fibrosis

Antibiotic strategies for eradicating Pseudomonas aeruginosa in people with CF

Antibiotic treatment for Burkholderia cepacia complex in people with cystic fibrosis experiencing a pulmonary exacerbation

Antibiotic treatment for non-tuberculous mycobacteria lung infection in people with cystic fibrosis

Antibiotic treatment for Stenotrophomonas maltophilia in people with cystic fibrosis

Antibiotics for treating acute chest syndrome in people with sickle cell disease

Antibiotics for treating community acquired pneumonia in people with sickle cell disease

Antibiotics for treating osteomyelitis in people with sickle cell disease

Antifungal therapies for allergic bronchopulmonary aspergillosis in people with CF

Anti-Ig E therapy for allergic bronchopulmonary aspergillosis in people with cystic fibrosis

Anti-inflammatory drugs and analgesics for managing symptoms in people with cystic fibrosis-related arthritis

Antioxidant supplementation for lung disease in cystic fibrosis

Appetite stimulants for people with cystic fibrosis

Bisphosphonate therapy for osteogenesis imperfecta

Bisphosphonates for osteoporosis in people with cystic fibrosis

Blood transfusion for preventing primary and secondary stroke in people with sickle cell disease

Blood transfusions for acute chest syndrome in people with sickle cell disease

Bronchoscopy-guided antimicrobial therapy for cystic fibrosis

Carnitine supplementation for treating people with inborn errors of metabolism

Chemical pleurodesis versus surgical intervention for persistent and recurrent pneumothoraces in cystic fibrosis

Chest physiotherapy versus no chest physiotherapy for cystic fibrosis

Clotting factor concentrates given to prevent bleeding & bleeding related complications in people with hemophilia A or B

Combination antimicrobial susceptibility testing for acute exacerbations in chronic infection of P. aeruginosa in CF

Conventional chest physiotherapy compared to other airway clearance techniques for CF

Deferasirox for iron chelation in people with transfusion-dependent thalassaemia

Deferasirox for managing transfusional overload in people with sickle cell disease

Desferrioxamine mesolate for managing transfusional iron overload in people with thalassaemia

Desmopressin acetate (DDAVP) for preventing acute bleeds during pregnancy in women with congenital bleeding disorders

Dietary advice with or without oral nutritional supplements for disease-related malnutrition in adults

Dietary interventions (plant sterols, stanols, omega-3 fatty acids, soy protein and barley) for familial hypercholesterolaemia

Dietary interventions for phenylketonuria

Disease-modifying anti-rheumatic drugs in people with cystic fibrosis-related arthritis

Dornase alfa for cystic fibrosis

Drug therapies for reducing gastric acidity in people with cystic fibrosis

Drugs for preventing red blood cell dehydration in people with sickle cell disease

Duration of IV antibiotic therapy for people with cystic fibrosis

Elective versus symptomatic intravenous antibiotics for cystic fibrosis

Embolisation for pulmonary arteriovenous malformation

Enteral tube feeding for cystic fibrosis

Enzyme replacement therapy for Anderson-Fabry disease

Enzyme replacement therapy with idursulfase for mucopolysaccharidosis type II (Hunter syndrome)

Enzyme replacement therapy with laronidase (Aldurazyme®) for treating mucopolysaccharidosis type I

Eradication therapy for Burkholderia cepacia complex (BCC) in people with cystic fibrosis

Fluid replacement therapy for acute episodes of pain in people with sickle cell disease

Gene therapy for hemophilia

Gene therapy for sickle cell disease

Haematopoietic stem cell transplantation for Gaucher disease

Haematopoietic stem cell transplantation for people with sickle cell disease

Hematopoietic stem cell transplantation for people with ß thalassaemia major

Home versus hospital intravenous antibiotic therapy for cystic fibrosis

Hydroxycarbamide for sickle cell disease

Immune tolerance induction for treating inhibitors in people with congenital haemophilia A or B

Immunosuppressive drug therapy to prevent rejection following lung transplantation for cystic fibrosis

Inhaled antibiotics for long-term therapy in cystic fibrosis

Inhaled antibiotics for pulmonary exacerbations in people with cystic fibrosis

Inhaled bronchodilators for acute chest syndrome in people with sickle cell disease

Inhaled bronchodilators for cystic fibrosis

Inhaled corticosteroids for cystic fibrosis

Inhaled nitric oxide for acute chest syndrome in people with sickle cell disease

Inspiratory muscle training for cystic fibrosis

Insulin and oral agents for managing cystic fibrosis-related diabetes

Interventions for promoting physical activity in people with cystic fibrosis

Interventions for reducing inflammation in familial Mediterranean fever

Interventions for the eradication of methicillin resistant Staphylococcus aureus in people with cystic fibrosis

Interventions for treating acute bleeding episodes in people with acquired hemophilia

Interventions for treating intrahepatic cholestasis in people with sickle cell disease

Interventions for treating leg ulcers in people with sickle cell disease

LMWHs for managing vaso-occlusive crises in people with sickle cell disease

Macrolide antibiotics for cystic fibrosis

Maternal and foetal outcomes following natural vaginal versus caesarean section (c-section) delivery in women with bleeding disorders and carriers

Nebulised hypertonic saline for cystic fibrosis

Nebuliser devices for drug delivery in cystic fibrosis

Nebulized and oral thiol derivatives for pulmonary disease in cystic fibrosis

Neonatal screening for sickle cell disease

Neuraminidase inhibitors for the treatment of influenza infection in people with cystic fibrosis

Newborn screening for cystic fibrosis

Newborn screening for homocystinuria

Non-invasive ventilation for cystic fibrosis

Non-surgical interventions for treating menorrhagia in women with bleeding disorders

Omega-3 fatty acids for cystic fibrosis

Once-daily vs multiple-daily dosing with intravenous aminoglycosides for cystic fibrosis

Oral anti-pseudomonal antibiotics for cystic fibrosis

Oral calorie supplements for cystic fibrosis

Oral deferiprone for iron chelation in thalassaemia

Oral non-steroidal anti-inflammatory drug therapy for lung disease in cystic fibrosis

Oral protein calorie supplementation for children with chronic disease

Oral steroids for long-term use in cystic fibrosis

Oscillating devices for airway clearance in people with cystic fibrosis

Oxygen therapy for cystic fibrosis

Palivizumab for prophylaxis against respiratory syncytial virus infection in children with cystic fibrosis

Pancreatic enzyme replacement therapy for people with cystic fibrosis

Percutaneous long lines for administering intravenous antibiotics in people with cystic fibrosis

Physical training for cystic fibrosis

Phytomedicines (medicines derived from plants) for sickle cell disease

Pine bark extract for the treatment of chronic disorders

Piracetam for reducing the incidence of sickle cell disease crises

Pneumococcal vaccines for cystic fibrosis

Pneumococcal vaccines for sickle cell disease

Positive expiratory pressure physiotherapy for airway clearance in people with CF

Potentiators (specific therapies for class III and IV mutations) for cystic fibrosis

Preoperative blood transfusions for sickle cell disease

Prophylactic antibiotics for preventing pneumococcal infection in people with sickle cell disease

Prophylactic anti-staphylococcal antibiotics for cystic fibrosis

Protein substitute for children and adults with phenylketonuria

Psychological interventions for individuals with cystic fibrosis and their families

Psychological therapies for sickle cell disease & pain

Psychological therapies for thalassaemia

Recombinant Factor VIIa concentrate vs plasma derived concentrates for the acute treatment of people with Haemophilia & inhibitors

Recombinant growth hormone therapy for children and young adults with cystic fibrosis

Recombinant growth hormone therapy for X-linked hypophosphatemia in children

Regular long-term red blood cell transfusions for chronic chest complications in sickle cell disease

Rituximab for treating inhibitors in people with inherited severe hemophilia

Sapropterin dihydrochloride for phenylketonuria

Self-management education for cystic fibrosis

Singing as an adjunct therapy for children and adults with cystic fibrosis

Single vs combination IV antibiotic therapy for people with cystic fibrosis

Sodium channel blockers for cystic fibrosis

Splenectomy vs conservative management for acute sequestration crises in people with sickle cell disease

Standard (head-down tilt) versus modified (without head-down tilt) postural drainage in infants and young children with cystic fibrosis

Standard versus biofilm antimicrobial susceptibility testing for infection of P. aeruginosa in CF

Statins for familial hypercholesterolemia in children

Surgical interventions for treating pectus excavatum

Timing of dornase alfa inhalation for cystic fibrosis

Timing of hypertonic saline inhalation in cystic fibrosis

Topical cystic fibrosis transmembrane conductance regulator gene replacement for cystic fibrosis-related lung disease

Topical nasal steroids for treating nasal polyposis in people with cystic fibrosis

Totally implantable vascular access devices for cystic fibrosis

Treatment for avascular necrosis of bone in people with sickle cell disease

Treatment for preventing bleeding in people with haemophilia or other congenital bleeding disorders undergoing surgery

Treatments for priapism in boys and men with sickle cell disease

Tyrosine supplementation for phenylketonuria

Ursodeoxycholic acid for cystic fibrosis-related liver disease

Vaccines for preventing infection with Ps. aeruginosa in people with cystic fibrosis

Vaccines for preventing influenza in people with cystic fibrosis

Vaccines for preventing invasive salmonella complications in people with sickle cell disease

Vitamin A supplementation for cystic fibrosis

Vitamin D supplementation for cystic fibrosis

Vitamin E supplementation in people with cystic fibrosis

Vitamin K supplementation for cystic fibrosis

Zinc supplementation for thalassaemia and sickle cell disease
