# Supplementary material for: The Use and Reporting of the Cross-Over Study Design in Clinical Trials and Systematic Reviews: A Systematic Assessment
Source: PLoS One. 2016 Jul 13;11(7):e0159014. doi: 10.1371/journal.pone.0159014 (PMC4943623; doi:10.1371/journal.pone.0159014)
Supplement: S2 File — Reference list of 218 unique cross-over trials included in Cochrane Cystic Fibrosis and Genetic Disorders reviews published to July 2015. (DOCX) [file pone.0159014.s002.docx]

**Supplementary File 2: Cross-over trials included in Cochrane Cystic Fibrosis and Genetic Disorders Group reviews.** Reference list of 218 unique cross-over trials included in Cochrane Cystic Fibrosis and Genetic Disorders reviews published to July 2015.

Adde FV, B. K., Hatanaka ACF, Nakaie CMA, Cardieri JMA, Oliveira RC, et al. (2004). "Hypertonic saline X recombinant human DNase: a randomised cross-over study in 18 cystic fibrosis patients [abstract]." Journal of Cystic Fibrosis 3(Suppl 1): S66.

Alvim RC, V. M., Pires MA, Franklin HM, Paula MJ, Brito AC, et al. (2005). "Inefficacy of piracetam in the prevention of painful crises in children and adolescents with sickle cell disease. ." Acta Haematologica 113(4): 228-233.

Amundsen AL, O. L., Nenseter MS, Ntanios FY. (2002). "Plant sterol ester-enriched spread lowers plasma total and LDL cholesterol in children with familial hypercholesterolemia." American Journal of Clinical Nutrition 76(2): 338-344.

Anderson P, M. J. (2009). "Evaluation of two different timings of Pulmozyme nebulisation in relation to chest physiotherapy in children with Cystic Fibrosis [abstract]." Journal of Cystic Fibrosis 8(Suppl 2): S74.

App EM, K. R., Reinhardt D, Lindemann H, Dasgupta B, King M, et al. (1998). "Sputum rheology changes in cystic fibrosis lung disease following two different types of physiotherapy: flutter vs autogenic drainage." Chest 114(1): 171.

Aronstam A, A. P., Rainsford SG, Turk P, Slattery M, Alderson MR, et al (1976). "Prophylaxis in hemophilia: a double-blind controlled trial." British Journal of Haematology 33(1): 81-90.

Aronstam A, K. P., McHardy J, Culver-James JW, McLEllan DS, Turk P, et al. (1977). "Twice weekly prophylactic therapy in haemophilia A." Journal of Clinical Pathology 30(1): 65-67.

Asher MI, P. R., Coates AL, Thomas E, Macklem PT. (1982). "The effects of inspiratory muscle training in patients with cystic fibrosis." American Review of Respiratory Disease 126(5): 855-859.

Assoufi BK, D. C., Hodson ME. (1994). "High dose Nutrizym 22 in adults with cystic fibrosis [abstract]." Pediatric Pulmonology 18(Suppl 10): 337.

Astermark J, D. S., DiMichele DM, Gringeri A, Gilbert SA, Waters J, et al. (2007). "A randomized comparison of bypassing agents in hemophilia complicated by an inhibitor: the FEIBA NovoSeven Comparative (FENOC) study." Blood 109(2): 546-551.

Avital A, S. I., Chernick V. (1992). "Efficacy of salbutamol and ipratropium bromide in decreasing bronchial hyperreactivity in children with cystic fibrosis. ." Pediatric Pulmonology 13(1): 34-37.

Balestri E, A. M., Dall’Ara S, Miano A. (2004). "Efficacy of physical exercise for mucus clearance in patients with cystic fibrosis (CF) [abstract]." Pediatric Pulmonology(Suppl 27): 316.

Balestrieri GP, M. V., Sleiman I, Spandrio S, Di Stefano O, Salvi A, et al. (1996). "Fish oil supplementation in patients with heterozygous familial hypercholesterolemia. ." Recenti Progressi in Medicina 87(3): 102-105.

Balfour-Lynn I, K. N. J., Dinwiddie R. (1997). "Randomised controlled trial of inhaled corticosteroids (fluticasone propionate) in cystic fibrosis." Archives of Disease in Childhood 77(2): 124-130.

Ballmann M, v. d. H. H. (2002). "Hypertonic saline and recombinant human DNase: a randomised cross-over pilot study in patients with cystic fibrosis." Journal of Cystic Fibrosis 1(1): 35-37.

Barker M, W. R., Hoffmann U, Ebfeld D, Kusenbach G. (1998). "Effects of hyperoxia on oxygen uptake kinetics in cystic fibrosis patients and healthy controls [abstract]. ." European Respiratory Journal 12(Suppl 28): 391s.

Battistini R, B. E., Ambroni M, Miano A. (2001). Efficacy of underwater positive expiratory pressure therapy (UPEP) for mucus clearance in patients with cystic fibrosis [abstract]. . Abstracts of the 24th European Cystic Fibrosis Conference

June 6-9; Vienna.

Beker LT, A. R., Fink RJ, O’Brien ME, Davidson KW, Sokoll LJ, et al (1997). "Effect of vitamin K1 supplementation on vitamin K status in cystic fibrosis patients." Journal of Pediatric Gastroenterology and Nutrition 24(5): 512-517.

Bilton D, D. M., Abbot JV, Webb AK (1992). "The benefits of exercise combined with physiotherapy in the treatment of adults with cystic fibrosis." Respiratory Medicine 86(6): 507-511.

Borgna-Pignatti C, C. A. (1997). "Evaluation of a new method of administration of the iron chelating agent deferoxamine." Journal of Pediatrics 130(1): 86-88.

Bowler IM, W. S., Littlewood JM, Chand R, McAllister J (1993). CF patients with significant malabsorption: effects of a high lipase enzyme preparation and ranitidine [abstract]. . Proceedings of the 18th European Cystic Fibrosis Conference, Madrid.

Boyle BJ, L. W., Balistreri WF, Widzer SJ, Huang N (1980). "Effect of cimetidine and pancreatic enzymes on serum and fecal bile acids and fat absorption in cystic fibrosis." Gastroenterology 78(5 pt 1): 950-953.

Braggion C, C. L., Cornacchia M, Zanolla L, Mastella G. (1995). "Short-term effects of three chest physiotherapy regimens in patients hospitalized for pulmonary exacerbations of cystic fibrosis: a cross-over randomized study. Pediatric Pulmonology 1995;19(1):16–22." Pediatric Pulmonology 19(1): 16-22.

Button BM, H. R., Catto-Smith AG, Olinsky A, Phelan PD, Ditchfield MR, et al. (2003). "Chest physiotherapy in infants with cystic fibrosis: to tip or not? A five-year study." Pediatric Pulmonology 35(3): 208-213.

Byrne NM, K. P., Gould FK, Spencer DA (2003). "Comparison of lung deposition of colomycin using the halolite and the Pari LC Plus nebulisers in patients with cystic fibrosis. ." Archives of Disease in Childhood 88(8): 715-718.

Carlsson M, B. E., Björkman S, Lethagen S, Ljung R. (1997). "Improved cost-effectiveness by pharmacokinetic dosing of factor VIIII in prophylactic treatment of haemophilia A. ." Haemophilia 3: 96-101.

Carroccio A, P. F., Montalto G, Iapichino L, Soresi M, Averna MR, et al. (1992). "Use of famotidine in severe exocrine pancreatic insufficiency with persistent maldigestion on enzymatic replacement therapy: A long term study in cystic fibrosis. ." Digestive Diseases and Sciences 37(9): 1441-1446.

Chadwick SL, M. S., Bott J, Geddes DM, Alton EWFW. (1997). "Effect of hypertonic , isotonic saline and water challenges on the airways of cystic fibrosis patients [abstract]. ." Thorax 52(Suppl 6): A43.

Chalmers DM, B. R., Miller MG, Clarke PCN, Kelleher J, Littlewood JM, et al. (1985). "The influence of long term cimetidine as an adjuvant to pancreatic enzyme therapy in cystic fibrosis. ." Acta Paediatrica Scandinavia 74(1): 114-117.

Chatham K, I. A., Nixon LS, Shale DJ. (2004). "A short-term comparison of two methods of sputum expectoration in cystic fibrosis." European Respiratory Journal 23(3): 435-439.

Chisholm A, S. W., Ball M. (1994). "The effects of dietary fat content on plasma noncholesterol sterol concentrations in patients with familial hypercholesterolaemia treated with simvastatin. ." Metabolism 43(3): 310-314.

Chung Y, G. T., Anst DB, Blue B, VanderBranden SV. (2000). "The impact of omeprazole on children with cystic fibrosis (CF) who require high dose pancreatic enzymes: A pilot study [abstract]." Journal of Pediatric Gastroenterology and Nutrition 31(Suppl 2): S73.

Clarke JTR, G. R., Hogan SE, Barrett M, MacDonald GW. (1987). "Neuropsychological studies on adolescents with phenylketonuria returned to phenylalanine-restricted diets. ." American Journal of Mental Retardation 92(3): 255–262.

Clavel A, B. A., Bosdure E, Luc C, Lanteaume A, Gorincour G, et al. Nebulisers comparison with inhaled tobramycin in young children with cystic fibrosis. Journal of Cystic Fibrosis 2007;6(2):137-43. (2007). "Nebulisers comparison with inhaled tobramycin in young children with cystic fibrosis." Journal of Cystic Fibrosis 6(2): 137-143.

Darbee J, D. S., Bensel K, Jehan A, Watkins M, Holsclaw D. (1990). "Radionuclide assessment of the comparative effects of chest physical therapy and positive expiratory pressure mask in cystic fibrosis [abstract]. ." Pediatric Pulmonology(Suppl 5): 251.

Darbee JC, K. J., Ohtake PJ. (2005). "Physiologic Evidence for high frequency chest wall oscillation and positive expiratory pressure breathing in hospitalized subjects with cystic fibrosis. ." Physical Therapy 85(12): 1278–1289.

Darbee JC, O. P., Grant BJB, Cerny FJ (2004). "Physiologic Evidence for the Efficacy of Positive Expiratory Pressure as an Airway Clearance Technique in Patients with Cystic Fibrosis." Physical Therapy 84(6): 524–537.

Davidson AGF, W. L., Pirie GE, McIlwaine PM. (1992). "Long-term comparative trial of conventional percussion and drainage physiotherapy versus autogenic drainage in cystic fibrosis [abstract]. ." Pediatric Pulmonology . 14(S8): 235.

Day AJ, W. J., McKeown C, Bruton A,Weller PH. Evaluation of inhaled colomycin in children with cystic fibrosis [abstract]. Proceedings of the 10th International Cystic Fibrosis Congress; 1988 March 5-10; Sydney. 1988:106. (1988). Evaluation of inhaled colomycin in children with cystic fibrosis [abstract]. . Proceedings of the 10th International Cystic Fibrosis Congress, March 5-10; Sydney.

Devadason SG, E. M., Linto JM, Le Souef PN. (1997). "Comparison of drug delivery from conventional versus "venturi" nebulisers. ." European Respiratory Journal 10(11): 2479-2483.

Devadason SG, H. T., Walker SL, Troedson R, Le Souef PN. (2001). Lung deposition of rhDNase in children children with cystic fibrosis using the Halolite adaptive aerosol delivery (AAD) systems and Pari LC+ nebulizer [abstract]. Proceedings of 24th European Cystic Fibrosis Conference, June 6-9 Vienna.

Dodd JD, B. S., Daly LE, Gallagher CG. Inhaled betaagonists improve lung function but not maximal exercise capacity in cystic fibrosis. Journal of Cystic Fibrosis 2005;4(2):101–5. (2005). "Inhaled betaagonists improve lung function but not maximal exercise capacity in cystic fibrosis. ." Journal of Cystic Fibrosis 4(2): 101-105.

Dodd ME, M. A., Haworth CS, Francis S, Miles, J, Clayton N, et al. (2000). The effect of rhDNase on exercise performance and gas trapping in adults with cystic fibrosis: a randomised controlled trial [abstract]. Proceedings of the 13th International Cystic Fibrosis Congress, June 4-8; Stockholm.

Doumit M, K. U., Jaffé A, Belessis Y. (2012). "Acid and nonacid reflux during physiotherapy in young children with cystic fibrosis." Pediatric Pulmonolgy 47(2): 119-124.

Duncan FR, P. A., Hodson ME, Batten JC. (1982). The use of bronchodilators as an adjunct to physiotherapy in adults with cystic fibrosis [abstract]. . Research Conference.

Durie PR, B. L., Linton W, Corey ML, Forstner GG. (1980). "Effect of cimetidine and sodium bicarbonate on pancreatic replacement therapy in cystic fibrosis. ." Gut 21(9): 778-786.

Edlund M, B. M., Fried G. (2002). "Desmopressin in the treatment of menorrhagia in women with no common coagulation factor deficiency but with prolonged bleeding time. ." Blood Coagulation and Fibrinolysis 13(3): 225-231.

Eggleston PA, R. B., Stackhouse CM, Mellitus ED, Baunmgardner RA. (1991). "A controlled trial of long-term bronchodilator therapy in cystic fibrosis. ." Chest 99(5): 1088-1092.

Eisenberg J, P. M., Williams-Warren J, Vasiliev M, Montgomery AB, Smith AL, et al. (1997). "A comparison of peak sputum concentration in patients with cystic fibrosis using jet and ultrasonic nebulizer systems." Chest 111(4): 955-962.

Elkins MR, B. P. (2006). "Comparison of Pari LC-Star and -Plus nebulisers delivering 2.5mg recombinant human deoxyribonuclease (rhDNase) [abstract]. ." Journal of Cystic Fibrosis 5(Suppl 1): S42.

Elkins MR, E. S., Constable C, White J, Robinson M, Daviskas E, et al. (2005). "The effect of manual chest physiotherapy, positive expiratory pressure (PEP), and oscillating PEP on mucociliary clearance in subjects with cystic fibrosis [abstract]. ." Pediatric Pulmonology 40(Suppl 28): 321.

Elliott RB, E. L., Lees HR, Akroyd RM, Reilly HC. (1992). "A comparison of two pancreatin microsphere preparations in cystic fibrosis." New Zealand Medical Journal 105(930): 107-108.

Engler MM, E. M., Malloy M, Chiu E, Besio D, Paul S, et al. (2004). "Docosahexaenoic acid restores endothelial function in children with hyperlipidemia: results from the EARLY Study. ." International Journal of Clinical Pharmacology and Therapeutics 42(12): 672-679.

Equi A, B.-L. I., Bush A, Rosenthal M. (2002). "Long term azithromycin in children with cystic fibrosis: a randomised, placebo-controlled crossover trial. ." Lancet 360(9338): 978-984.

Fainardi V, L. F., Faverzani S, Tripodi MC, Chetta A, Pisi G (2011). "Short-term effects of high-frequency chest compressions and positive expiratory pressure in patients with cystic fibrosis." Journal of Clinical Medicine Research 3(6): 279–284.

Falk B, N. A., Zigel L, Yahav Y, Aviram M, Rivlin J, et al. (2006). "Effect of low altitude at the Dead Sea on exercise capacity and cardiopulmonary response to exercise in cystic fibrosis patients with moderate to severe lung disease." Pediatric Pulmonology 41(3): 234-241.

Falk M, K. M., Andersen JB, Kinoshita T, Falk P, Stovring S, et al. (1984). "Improving the ketchup bottle method with positive expiratory pressure, PEP, in cystic fibrosis. ." European Journal of Respiratory Diseases 65(6): 423-432.

Falk M, M. J., Kelstrup M, Lanng S, Larsen L, Ulrik CS. (1993). "Short-term effects of positive expiratory pressure and the forced expiration technique on mucus clearance and lung function in CF [abstract]. ." Pediatric Pulmonology(Suppl 9): 241.

Fauroux B, B. M., Lofaso F, Zerah F, Clement A, Harf A, et al. (1999). "Chest physiotherapy in cystic fibrosis: improved tolerance with nasal pressure support ventilation. ." Pediatrics 103(3): E32.

Ferster A, V. C., Cornu G, Buyse M, Corazza F, Devalck C, et al. (1996). "Hydroxyurea for treatment of severe sickle cell anemia: a pediatric clinical trial. ." Blood 88(6): 1960-1964.

Fitzgerald DA, H. J., Jepson B, Smith L. (2005). "A crossover, randomized, controlled trial of dornase alfa before versus after physiotherapy in cystic fibrosis." Pediatrics 116(4): e549–554.

Francisco MP, W. M., Sherman JM, Theriiaque D, Bowser E, Novak DA. (2002). "Ranitidine and Omeprazole as adjuvant therapy to pancrealipase to improve fat absorption in patients with cystic fibrosis." Journal of Pediatric Gastroenterology and Nutrition 35(1): 79-83.

Geller DE, R. M., Waltz DA, Wilmott RW. (2003). "Efficiency of pulmonary administration of tobramycin solution for inhalation in cystic fibrosis using an improved drug delivery system." Chest 123(1): 28-36.

Giles D, S. M., Wagener J, Accurso F. (1996). "Effect of one month of treatment with flutter valve or postural drainage and clapping on pulmonary function and sputum recovery in cystic fibrosis [abstract]. ." Pediatric Pulmonology(Suppl 13): 307.

Goldstein RC, S. A. (1974). "Prophylactic colchicine therapy in familial Mediterranean fever. A controlled, double-blind study." Annals of Internal Medicine 81(6): 792-794.

Gotz M, W. A. (1995). "Physiotherapy in cystic fibrosis: Intrapulmonary percussive ventilation (IPV) versus positive expiratory pressure (PEP) [abstract]. ." Pediatric Pulmonology 20(Suppl 12): 267.

Gozal D (1997). "Nocturnal ventilatory support in patients with cystic fibrosis: comparison with supplemental oxygen." European Respiratory Journal 10(9): 1999-2003.

Graham A, H. A., Alton EWFW, Martin GP, Marriott C, Hodson ME, et al. (1993). "No added benefit from nebulized amiloride in patients with cystic fibrosis." European Respiratory Journal 6(9): 1243-1248.

Grieve R, T. S., Normand C, Suri R, Bush A,Wallis C. (2003). "A cost-effectiveness analysis of rhDNase in children with cystic fibrosis." International Journal of Technology Assessment in Health Care 19(1): 71-79.

Griffiths P, W. N., Harvie A, Cockburn F. (1998). "Neuropsychological outcome of experimental manipulation of phenylalanine intake in treated phenylketonuria. ." Journal of Inherited Metabolic Disease 21(1): 29-38.

Grover P, T. W., Moran A. (2008). "Glargine versus NPH insulin in cystic fibrosis related diabetes. ." Journal of Cystic Fibrosis 7(2): 134-136.

Guardamagna O, A. F., Baracco V, Stasiowska B, Martino F. (2011). "The treatment of hypercholesterolemic children: efficacy and safety of a combination of red yeast rice extract and policosanols. ." Nutrition, Metabolism and Cardiovascular Diseases 21(6): 424-429.

Gylling H, S. M., Miettinen TA. (1995). "Sitostanol ester margarine in dietary treatment of children with familial hypercholesterolemia. ." Journal of Lipid Research 36: 1807-1812.

Hansen LG, W. W. (1990). "High-frequency chest compression system to aid in clearance of mucus from the lung. ." Biomedical Instrumentation & Technology 24(4): 289-294.

Hashkes PJ, S. S. J., Giannini EH, Huang B, Johnson A, Park G , et al (2012). "Rilonacept for colchicine-resistant or - intolerant familial Mediterranean fever: a randomized trial." Annals of Internal Medicine 157(8): 533-541.

Hayes J, C. C., O’Connor C, FitzGerald MX. (1995). "The effect of high dose inhaled corticosteroids on levels of sputum or plasma elastase IL-8 in patients with mild cystic fibrosis." Irish Journal of Medical Science 164(2): 165.

Heijerman HGM, L. C., Dijkman JH, Bakker W. (1990). "Ranitidine compared with the dimethylprostagalndin E2 analogue enprostil as adjunct to pancreatic enzyme replacement in adult cystic fibrosis. ." Scandinavian Journal of Gastroenterology 178(Suppl): 26-31.

Heijerman HGM, L. C., Bakker W. (1991). "Omeprazole enhances the efficacy of pancreatin (pancrease) in cystic fibrosis. ." Annals of Internal Medicine 114(3): 200-201.

Heijerman HGM, L. C., Bakker W, Dijkman JH. (1993). "Improvement of fecal fat excretion after addition of omeprazole to pancrease in cystic fibrosis is related to residual exocrine function of the pancreas. ." Digestive Diseases and Sciences 31(1): 1-6.

Henker J, J. H. L., Vogt E. (1987). Comparison of effectiveness of Pankreon ForteR and KreonR in children with cystic fibrosis [abstract]. . 15th Annual Meeting of the European Working Group for Cystic Fibrosis, Oslo, Norway.

Hodson ME, P. A., Batten JC. (1981). "Aerosol carbenicillin and gentamicin treatment of Pseudomonas aeruginosa infection in patients with cystic fibrosis. ." Lancet 2(8256): 1127-1129.

Hofmeyr JL, W. B., Hodson ME. (1986). "Evaluation of positive expiratory pressure as an adjunct to chest physiotherapy in the treatment of cystic fibrosis. ." Thorax 41(12): 951-954.

Holland AE, D. L., Ntoumenopoulos G, Naughton MT, Wilson JW. (2003). "Non-invasive ventilation assists chest physiotherapy in adults with acute exacerbations of cystic fibrosis. ." Thorax 58(10): 880-884.

**Homnick DN, A. K., Marks JH. (1998). "Comparison of the flutter device to standard chest physiotherapy in hospitalized patients with cystic fibrosis: a pilot study." Chest 114(4): 993-997.*

Hordvik NL, S. P., Judy CG, Strizek SJ, Colombo JL. (1996). "The effects of albuterol on the lung function of hospitalized patients with cystic fibrosis." American Journal of Respiratory and Critical Care Medicine 154(1): 156-160.

Hordvik NL, S. P., Judy CG, Colombo JL. (1999). "Effects of standard and high doses of salmeterol on lung function of hospitalized patients with cystic fibrosis. ." Pediatric Pulmonolgy 27(1): 45-53.

Hordvik NL, S. P., Judy CG, Colombo JL. (2002). "Effectiveness and tolerability of high dose salmeterol in cystic fibrosis. ." Pediatric Pulmonolgy 34(4): 287-296.

Hosseini S, L. J., Sepulveda RT, Rohdewald P, Watson RR. (2001). "A randomized, double-blind, placebo-controlled, prospective, 16 week crossover study to determine the role of Pycnogenol in modifying blood pressure in mildly hypertensive patients. ." Nutrition Research 21(9): 1251-1260.

Hosseini S, P. S., Sadrzadeh SMH, Farid F, Farid R, Watson RR. (2001). "Pycnogenol® in the management of asthma. ." The Journal of Medicinal Food 4(4): 201-209.

Howard J, B. J., Hewitt O, Elborn S. (2000). "The active cycle of breathing (ACBT) is a more effective method of airway clearance in cystic fibrosis (CF) patients than the test of incremental respiratory endurance (TIRE) [abstract]. ." Pediatric Pulmonology 30(Suppl 20): 304.

Howatt WF, D. G. (1966). "A double-blind study of the use of acetylcysteine in patients with cystic fibrosis." University of Michigan Medical Center Journal 32(2): 82-85.

Hubert D, L. S., Nove-Josserand R, Murris-Espin M, Mely L, Dominique S, et al. (2009). "Pharmacokinetics and safety of tobramycin administered by the PARI eFlow rapid nebulizer in cystic fibrosis. ." Journal of Cystic Fibrosis 8(5): 332-337.

Hütler M, S. D., Staab D, Tacke A, Wahn U, et al (2002). "Effect of growth hormone on exercise tolerance in children with cystic fibrosis." Medicine and Science in Sports and Exercise 34(4): 567-572.

Jarad NA, P. T., Smith E. (2010). "Evaluation of a novel sputum clearance technique--hydro-acoustic therapy (HAT) in adult patients with cystic fibrosis: a feasibility study. ." Chronic Respiratory Diseases 7(4): 217-227.

Kadir RA, L. C., Sabin CA, Pollard D, Economides DL. (2002). "DDAVP nasal spray for treatment of menorrhagia in women with inherited bleeding disorders: a randomized placebo-controlled crossover study." Haemophilia 8(6): 787-793.

Kastelik JA, W. G., Aziz A, Davies M, Avery GR, Paddon AJ, et al. (2002). "A widely available method for the assessment of aerosol delivery in cystic fibrosis. ." Pulmonology Pharmacology & Therapeutics 15(6): 513-519.

Ketomäki A, G. H., Miettinen TA. (2004). "Removal of intravenous Intralipid in patients with familial hypercholesterolemia during inhibition of cholesterol absorption and synthesis. ." Clinica Chimica Acta 344(1-2): 83-93.

Ketomäki A, G. H., Miettinen TA. (2005). "Non-cholesterol sterols in serum, lipoproteins and red cells in statin-treated familial hypercholesterolemia subjects off and on plant stanol and sterol ester spreads. ." Clinica Chimica Acta 353(1-2): 75-86.

Ketomaki AM, G. H., Antikaimen M, Simes MA, Miettinen TA. (2003). "Red cell and plasma plant sterols are related during consumption of plant sterols are related during consumption of plant stanol ester spreads in children with hypercholesterolemia. ." Journal of Pediatrics 142(5): 524-531.

Kluft J, B. L., Castagnino M, Gaiser J, Chaney H, Fink RJ. (1996). "A comparison of bronchial drainage treatments in cystic fibrosis. ." Pediatric Pulmonology 22(4): 271-274.

Knowles MR, C. N., Waltner WE, Yankaskas JR, Gilligan P, King M, et al. (1991). "Aerosolized amiloride as treatment of cystic fibrosis lung disease: a pilot study. ." Advances in Experimental Medicine and Biology 290: 119-128.

Kofler AM, B. M., Bressan T, Carlesi A, Leone P, Lucidi V, et al. (1994). PEP-mask and active cycle of breathing techniques. What is better in children with cystic fibrosis [abstract].

. Proceedings of 19th European Cystic Fibrosis Conference, May 29-June 3; Paris, France.

Kofler AM, C. A., Cutrera R, Leone P, Lucidi V, Rosati S, et al. (1998). "BiPAP versus PEP as chest physiotherapy in patients with cystic fibrosis [abstract]. ." Pediatric Pulmonology 26(Suppl 17): 344.

Köhler E, S. V., Schuster-Wonka R, Hühnerbein J. (2003). "Lung deposition in cystic fibrosis patients using an ultrasonic or a jet nebuliser. ." Journal of Aerosol Medicine 16(1): 37-46.

Konig P, P. J., Barbero GJ. (1998). "A placebo-controlled double-blind trial of the long-term effectsof albuterol administration in patients with cystic fibrosis. ." Pediatric Pulmonology 25(1): 32-36.

Kouides PA, B. R., Philipp CS, Stein SF, Heit JA, et al. (2009). "Multisite management study of menorrhagia with abnormal laboratory haemostasis: a prospective crossover study of intranasal desmopressin and oral tranexamic acid." British Journal of Haematology 145(2): 212-220.

Kraig R, K. K., Howard D, Ter-Pogossian M, Kollef MH. (1995). "A direct comparison of manual chest percussion with acoustic percussion, an experimental treatment for cystic fibrosis [abstract]. ." American Journal of Respiratory and Critical Care Medicine Supplements 151(4): A738.

Kun P, L. L., Phelan PD. (1984). "Nebulized gentamicin in children and adolescents with cystic fibrosis." Australian Paediatric Journal 20(1): 43-45.

Lacy D, W. J., Venkataraman M, Vyas J, MacDonald A, Weller PH, et al. (1992). A comparison of Nutrizym GR and Creon in children with CF [abstract]. . 11th International Cystic Fibrosis Congress, Dublin, Ireland.

Lagerkvist AL, S. G., Redfors SB, Lindblad AG, Hjalmarson O. (2006). "Immediate changes in blood-gas tensions during chest physiotherapy with positive expiratory pressure and oscillating positive expiratory pressure in patients with cystic fibrosis." Respiratory Care 51(10): 1154-1161.

Lannefors L, W. P. (1992). "Mucus clearance with three chest physiotherapy regimes in cystic fibrosis: a comparison between postural drainage, PEP and physical exercise. ." European Respiratory Journal 5(6): 748-753.

Laurin D, J. H., Moorjani S, Steinke F, Gagne C, Brun D, et al. (1991). "Effects of a soy-protein beverage on plasma lipoproteins in children with familial hypercholesterolemia. ." American Journal of Clinical Nutrition 54: 98-103.

Lawrence R, S. T. (1993). "Eicosapentaenoic acid in cystic fibrosis: evidence of a pathogenetic role for leukotriene B4. ." Lancet 342(8869): 465-469.

Ledson MJ, G. M., Robinson M, Cowperthwaite C, Willets T, Hart CA, Walshaw MJ. (2002). "A randomized doubleblinded placebo-controlled crossover trial of nebulized taurolidine in adult cystic fibrosis patients infected with Burkholderia cepacia. ." Journal of Aerosol Medicine 15(1): 51-57.

Lenney W, E. F., Kho P, Kovarik JM. (2011). "Lung deposition of inhaled tobramycin with eFlow rapid/LC plus jet nebuliser in healthy and cystic fibrosis subjects. ." Journal of Cystic Fibrosis 10(1): 9-14.

Lyons E, C. K., Campbell IA, Prescott RJ. (1992). Evaluation of the flutter VRP1 device in young adults with cystic fibrosis [abstract]. . 11th International Cystic Fibrosis Congress, Dublin, Ireland.

MacDonald A, C. A., Hendriksz C, Daly A, Davies P, Asplin D, et al. (2006). "Protein substitute dosage in PKU: how much do young patients need?" Archives of Disease in Childhood 91(7): 588-593.

Manguso F, D. A. G., Menchise A, Sollazzo R, D’Agostino L. (2005). "Effects of an appropriate oral diet on the nutritional status of patients with HCV-related liver cirrhosis: a prospective study. ." Clinical Nutrition 24(5): 751–759.

Marchand V, B. S., Stark TJ, Baker RD (2000). "Randomized, double-blind, placebo-controlled pilot trial of megestrol acetate in malnourished children with cystic fibrosis." Journal of Pediatric Gastroenterology and Nutrition 31(3): 264-269.

Marcus CL, B. D., Stabile MW, Wang C-I, Osher AB, Keens TG. (1992). "Supplementation oxygen and exercise performance in patients with cystic fibrosis with severe pulmonary disease. ." Chest 101(1): 52-57.

Marshall LM, F. P., Khafagi FA. (1994). "Aerosol deposition in cystic fibrosis using an aerosol conservation device and a conventional jet nebuliser. ." Journal of Paediatrics and Child Health 30(1): 65-67.

Mazzocco MM, Y. S., Nord AM, Van Doorninck W, Davidson-Mundt AJ, Greene CL. (1992). "Cognition and tyrosine supplementation among school-aged children with phenylketonuria. ." American Journal of Diseases of Children 146(11): 1261-1264.

McIlwaine PM, D. A. (1991). Comparison of positive expiratory pressure and autogenic drainage with conventional percussion and drainage therapy in the treatment of cystic fibrosis [abstract]. Proceedings of the 17th European Cystic Fibrosis Conference, June 18-21; Copenhagen, Denmark.

McKone EF, B. S., FitzGeraldMX, Gallagher CG. (2002). "The role of supplemental oxygen during submaximal exercise in patients with cystic fibrosis. ." European Respiratory Journal 20(1): 134-142.

Merli M, B. S., Servi R, Diamanti S, Martino F, De Santis A, et al (1994). "Effect of a medium dose of ursodeoxycholic acid with or without taurine supplementation on the nutritional status of patients with cystic fibrosis. ." Journal of Pediatric Gastroenterology and Nutrition 19(2): 198-203.

Middleton PG, B. J. (2001). "Dornase alpha and physiotherapy - which should be first? A randomised, double-blind, placebo-controlled trial in CF adults [abstract]. ." Pediatric Pulmonology 32(Suppl 22): 310.

Mikati MA, S. H., Deryan DE, Sahli IF, Dabbous IA. (1983). "A preliminary report on piracetam in sickle cell anemia: a doubleblind crossover clinical trial and effects on erythrocyte survival." The King Faisal Specialist Hospital Medical Journal 3(4): 233-236.

Miller S, H. D., Clayton CB, Nelson R. (1995). "Chest physiotherapy in cystic fibrosis: a comparative study of autogenic drainage and the active cycle of breathing techniques with postural drainage." Thorax 50(2): 165-169.

Milne SM, E. C. (2004). "A pilot study comparing two physiotherapy techniques in patients with cystic fibrosis. ." South African Journal of Physiotherapy 60(2): 3-6.

Milross MA, P. A., Norman M, Becker HF, Willson GN, Grunstein RR, et al. (2001). "Low-flow oxygen and bilevel ventilator support. ." American Journal of Respiratory and Critical Care Medicine 163(1): 129-134.

Mitchell EA, E. R. (1982). "Controlled trial of oral N-acetylcysteine in cystic fibrosis. ." Australian Paediatric Journal 18(1): 40-42.

Moran A, P. J., Milla C. (2001). "Insulin and glucose excursion following pre-meal insulin lispro or repaglinide in cystic fibrosis-related diabetes. ." Diabetes Care 24(10): 1706-1710.

Morfini M, M. P., Mariani G, Panicucci F, Petrucci F, Baicchi U, et al. (1976). "Evaluation of prophylactic replacement therapy in haemophilia B. ." Scandinavian Journal of Haematology 16(1): 41-47.

Mortensen J, F. M., Groth S, Jensen C. (1991). "The effects of postural drainage and positive expiratory pressure physiotherapy on tracheobronchial clearance in cystic fibrosis." Chest 100(5): 1350-1357.

Nathanson I, C. G., Li P, Neter P. (1985). "Effectiveness of aerosolized gentamicin in cystic fibrosis (CF) [abstract]. ." Cystic Fibrosis Club Abstracts 28: 145.

Neil HA, M. G., Roe LS. (2001). "Randomised controlled trial of use by hypercholesterolaemic patients of a vegetable oil sterol-enriched fat spread. ." Atherosclerosis 156(2): 329-327.

Newman SP, W. G., Clarke SW. (1988). "Deposition of carbenicillin aerosols in cystic fibrosis: effects of nebuliser system and breathing pattern. ." Thorax 43(4): 318-322.

Nigon F, S.-L. C., Beucler I, Chauvois D, Neveu C, Giral P, et al. (2001). "Plant sterol-enriched margarine lowers plasma LDL in hyperlipidemic subjects with low cholesterol intake: effect of fibrate treatment. ." Clinical Chemistry and Laboratory Medicine 39(7): 634-640.

Nikolaizik WH, V. D., Ratjen F. (2008). "A pilot study to compare tobramycin 80 mg injectable preparation with 300 mg solution for inhalation in cystic fibrosis patients. ." Canadian Respiratory Journal 15(5): 259-262.

Nixon PA, O. D., Curtis SE, Ross EA. (1990). "Oxygen supplementation during exercise in cystic fibrosis. ." American Review of Respiratory Disease 142(4): 807-811.

O’Connor BS, R. A., Steen HJ, Shields MD, Elborn JS. (2008). "Use of azithromycin in cystic fibrosis patients not infected with Pseudomonas aeruginosa [abstract]. ." Journal of Cystic Fibrosis 7(Suppl 2): S25.

Oermann CM, S. M., Giles D, Sontag MK, Accurso FJ, Castile RG. (2001). "Comparison of high-frequency chest wall oscillation and oscillating positive expiratory pressure in the home management of cystic fibrosis: a pilot study. ." Pediatric Pulmonology 32(5): 372-377.

Olivieri NF, K. G., Hermann C, Bentur Y, Chung D, Klein J, et al. (1990). "Comparison of oral iron chelator L1 and desferrioxamine in iron-loaded patients. ." Lancet 336(8726): 1275-1279.

Osman.L.P, R. M., Hodson. M.E, Pryor.J.A. (2008). "High frequency chest wall oscillation in cystic fibrosis. ." Journal of cystic fibrosis. 7(Suppl 2): 295.

Padman R, G. D., Engelhardt MT. (1999). "Effects of the flutter device on pulmonary function studies among pediatric cystic fibrosis patients. ." Delaware Medical Journal 71(1): 13-18.

Panchaud A, S. A., Kernan Y, Decosterd LA, Buclin T, Boulat O, et al. (2006). "Biological effects of a dietary omega-3 polyunsaturated fatty acids supplementation in cystic fibrosis patients: A randomised, crossover placebo-controlled trial. ." Clinical Nutrition 25(3): 418-427.

Parsons SW, G. R., Torzillo PJ, Sullivan CE, Bye PTP. (1996). "The effects of nocturnal low-flow oxygen (nLFO2) on sleep function and gas exchange in patients with cystic fibrosis (CF) [abstract]. ." American Journal of Respiratory and Critical Care Medicine 153(Suppl): A72.

Patchell CJ, D. M., Weller PH, MacDonald A, Smyth RL, Bush A, et al. (2002). "Creon® 10000 minimicrospheres™ vs. Creon® 8000 microspheres - an open randomised crossover preference study. ." Journal of Cystic Fibrosis 1(4): 287-291.

Pedersen SS, P. T., Pedersen M, Hoiby N, Friis-Moller A, Kock C. (1986). "Immediate and prolonged clinical efficacy of Ceftazidime versus Ceftazidime plus Tobramycin in chronic Pseudomonas aeruginosa infection in cystic fibrosis. ." Scandinavian Journal of Infectious Diseases 18(133-7).

Petersen W, H. C., Garne S. (1987). "Pancreatic enzyme supplementation as acid-resistant microspheres versus enteric-coated granules in cystic fibrosis. A double placebo- controlled cross-over study. ." Acta Paediatrica Scandinavica 76(1): 66-69.

Pfleger A, T. B., Oberwaldner B, Zach MS. (1992). "Self administered chest physiotherapy in cystic fibrosis: a comparative study of high-pressure PEP and autogenic drainage. ." Lung 170(6): 323-330.

Phillips GE, P. S., Jaffe A, Bush A. (2004). "Comparison of active cycle of breathing and high-frequency oscillation jacket in children with cystic fibrosis." Pediatric Pulmonology 37(1): 71-75.

Pietz J, L. R., Kutscha A, Schmidt H, de Sonneville L, Trefz FK. (1995). "Effect of high-dose tyrosine supplementation on brain function in adults with phenylketonuria." Journal of Pediatrics 127(6): 936-943.

Pike SE, M. A., Dix KJ, Pryor JA, Hodson ME. (1999). "Comparison of flutter VRPI and forced expirations (FE) with active cycle of breathing techniques (ACBT) in subjects with cystic fibrosis (CF) [abstract]. ." The Netherlands Journal of Medicine 54(Suppl): S55-56.

Pitcher-Wilmott R, M. D., Ingram D, Tyson SL. (1982). Improvement in lung function after nebulised salbutamol and ipratropium in children with cystic fibrosis [abstract]. 11th European Cystic Fibrosis Conference.

Placidi G, C. M., Polese G, Zanolla L, Assael B, Braggion C. (2006). "Chest physiotherapy with positive airway pressure: a pilot study of short-term effects on sputum clearance in patients with cystic fibrosis and severe airway obstruction. ." Respiratory Care 51(10): 1145-1153.

Popescu M, M. J., Hillman L. (1998). "Calcium and vitamin D supplementation in CF children [abstract]. ." Pediatric Pulmonology 26(Suppl 17): 359.

Portal B, R., Coudray C, Arnaud J, Favier A. (1995). "Effect of double-blind cross-over selenium supplementation on lipid peroxidation markers in cystic fibrosis patients." Clinica Chimica Acta (International Journal of Clinical Chemistry) 1995(34): 1-2.

Prasad AS, A. A., Rabbani P, Dumouchelle E (1981). "Effect of zinc supplementation on serum testosterone level in adult male sickle cell anemia subjects." American Journal of Hematology 10(2): 119-127.

Proesmans M, D. B. K. (2003). "Omeprazol, a proton pump inhibitor, improves residual steatorrhoea in cystic fibrosis patients treated with high dose pancreatic enzymes. ." European Journal of Pediatrics 162(11): 760-763.

Pryor JA, W. B., Hodson ME, Batten JC. (1979). "Evaluation of the forced expiration technique as an adjunct to postural drainage in treatment of cystic fibrosis. ." BMJ 2(6187): 417-418.

Pryor JA, W. B., Hodson ME, Warner JO. (1994). "The Flutter VRP1 as an adjunct to chest physiotherapy in cystic fibrosis. ." Respiratory Medicine 88(9): 677-681.

Ramsey BW, D. H., Eisenberg JD, Gibson RL, Harwood IR, Kravitz RM, et al. (1993). "Efficacy of aerosolized tobramycin in patients with cystic fibrosis. ." New England Journal of Medicine 328(24): 1740-1746.

Riedler J, R. T., Button B, Robertson CF. (1996). "Inhaled hypertonic saline increases sputum expectoration in cystic fibrosis. ." Journal of Paediatric Child Health 32: 48-50.

Riethmueller J, B. M., Schroeter TW, Franke P, von Butler R, Claass A, et al. (2009). "Tobramycin once- vs thricedaily for elective intravenous antipseudomonal therapy in pediatric cystic fibrosis patients. ." Infection 37(5): 424-431.

Rigau-Perez JG, O. G., Chan LS,Weiss J, Powars D (1983). "Reactions to booster pneumococcal vaccination in patients with sickle cell disease." Pediatric Infectious Disease 2(3): 199–202.

Robinson M, D. E., Eberl S, Baker J, Chan H, Anderson, S, et al. (1999). "The effect of inhaled mannitol on bronchial mucus clearance in cystic fibrosis patients: a pilot study. ." European Respiratory Journal 14(3): 678–685.

Robinson M, H. A., Regnis J, Wong A, Bailey D, Bautotvich G, et al. (1997). "Effect of increasing doses of hypertonic saline on mucociliary clearance inpatients with cystic fibrosis. ." Thorax 52(10): 900-903.

Robinson M, R. J., Bailey DL, King M, Bautovich G, Bye PTP. (1996). "The effects of hypertonic saline, amiloride and cough on mucociliary clearance in patients with cystic fibrosis. ." American Journal of Respiratory and Critical Care Medicine 153(5): 1503-1509.

Robinson PJ, S. P., Smith AL. (1988). "Effect of misoprostol on fat malabsorption in cystic fibrosis. ." Archives of Disease in Childhood 63(9): 1081-1082.

Robinson PJ, S. P. (1990). "Placebo-controlled trial of misoprostol in cystic fibrosis. ." Journal of Pediatric Gastroenterology and Nutrition 11(1): 37-40.

Rossman CM, W. R., Sampson D, Newhouse MT. (1982). "Effect of chest physiotherapy on the removal of mucus in patients with cystic fibrosis. ." American Review ff Respiratory Disease 126(1): 131-135.

Salvatore D, D. A. M. (2002). "Effects of salmeterol on arterial oxyhaemoglobin saturations in patients with cystic fibrosis. Pediatric Pulmonology 2002;34(1):11–5." Pediatric Pulmonology 34(1): 11-15.

Sanchez I, D. K. J., Holbrow J, Chernick V. (1993). "The effect of high doses of inhaled salbutamol and ipratropium bromide in patients with stable cystic fibrosis. ." Chest 104(3): 842-846.

Sanchez I, H. J., Chernick V. (1992). "Acute bronchodilator response to a combination of beta-adrenergic and anticholinergic agents in patients with cystic fibrosis. ." Journal of Pediatrics 120(3): 486-488.

Schindeler S, G.-J. S., Thompson S, Rocca A, Joy P, Kemp A, et al. (2007). "The effects of large neutral amino acid supplements in PKU: an MRS and neuropsychological study. ." Molecular Genetics and Metabolism 91(1): 48-54.

Seikaly MG, B. R., Baum M. (1997). "The effect of recombinant human growth hormone in children with X-linked hypophosphatemia. ." Pediatrics 100(5): 879-884.

Seikaly MG, K. S., Salhab N, Waber P, Patterson D, Browne R, et al. (2005). "Impact of alendronate on quality of life in children with osteogenesis imperfecta. ." Journal of Pediatric Orthopaedics 25(6): 786-791.

Serisier DJ, C. A., Bowler SD. (2007). "Effect of albuterol on maximal exercise capacity in cystic fibrosis. ." Chest 131(4): 1181-1187.

Serjeant GR, d. C. K., Maude GH. (1985). "Stilboestrol and stuttering priapism in homozygous sickle-cell disease. ." Lancet 2(8467): 1274.

Shah AR, K. T., Gozal D. (1997). "Effect of supplemental oxygen on supramaximal exercise performance and recovery in cystic fibrosis. ." Journal of Applied Physiology 83(5): 1641-1647.

Smith ML, H. W., Clarke JTR, Klim P, SchoonheytW, Austin V, et al. (1998). "Randomised controlled trial of tyrosine supplementation on neuropsychological performance in phenylketonuria." Archives of Disease in Childhood 78(2): 116-121.

Spier S, R. J., Hughes D, Levison H. (1984). "The effect of oxygen on sleep, blood gases, and ventilation in cystic fibrosis. ." The American Review of Respiratory Disease 129: 712-718.

Stafanger G, G. S., Howitz P, Morkassel E, Koch C. (1988). "The clinical effect and the effect on the ciliary motility of oral Nacetylcysteine in patients with cystic fibrosis and primary ciliary dyskinesia. ." European Respiratory Journal 1(2): 161-167.

Stafanger G, K. C. (1989). "N-acetylcysteine in cystic fibrosis and Pseudomonas aeruginosa infection: clinical score, spirometry and ciliary motility. ." European Respiratory Journal 2(3): 234-237.

Stead RJ, H. M., Batten JC. (1987). "Inhaled ceftazidime compared with gentamicin and carbenicillin in older patients with cystic fibrosis infected with Pseudomonas aeruginosa. ." British Journal of Diseases of the Chest 81(3): 272-279.

Stead RJ, S. I., Hodson ME, Batten JC. (1987). "Enteric coated microspheres of pancreatin in the treatment of cystic fibrosis: comparison with a standard enteric coated preparation. ." Thorax 42(7): 533-537.

Stead RJ, S. I., Hodson ME. (1988). "Treatment of steatorrhoea in cystic fibrosis: a comparison of enteric-coated microspheres of pancreatin versus non-enteric-coated pancreatin and adjuvant cimetidine. ." Alimentary Pharmacology & Therapeutics 2(6): 471-482.

Steen HJ, R. A., O’Neill D, Beattie F. (1991). "Evaluation of the PEP mask in cystic fibrosis. ." Acta Paediatrica Scandinavica 80(1): 51-56.

Steven MH, P. J., Webber BA, Hodson MR. (1992). "Physiotherapy versus cough alone in the treatment of cystic fibrosis. ." New Zealand Journal Physiotherapy 20(2): 31-37.

Thomas SH, O. D. M., Graham A, Page CJ, Blower P, Geddes DM, et al. (1991). "Pulmonary deposition of nebulised amiloride in cystic fibrosis:comparison of two nebulisers. ." Thorax 46(10): 717-721.

Turchetta A, L. V., Bella S, Rosati P, Pastore E, Giordano U, et al. (1996). "Is salmeterol effective in children with cystic fibrosis [abstract]. ." Israel Journal of Medical Sciences 32(Suppl): S191.

Tyrrell JC, H. E., Martin J. (1986). "Face mask physiotherapy in cystic fibrosis." Archives of Disease in Childhood 61(6): 598-600.

van Asperen PP, J. L., Hennessy P, Brown J. (1987). "Comparison of a positive expiratory pressure (PEP) mask with postural drainage in patients with cystic fibrosis." Australian Journal of Paediatrics 23(5): 283-284.

van der Giessen LJ, d. J. J., Gosselink R, HopWC, Tiddens HA. (2007). "RhDNase before airway clearance therapy improves airway patency in children with CF. ." Pediatric Pulmonology 42(7): 624-630.

van der Giessen LJ, G. R., Hop WC, Tiddens HA. (2007). "Recombinant human DNase nebulisation in children with cystic fibrosis: before bedtime or after waking up?" European Respiratory Journal 30(4): 763-768.

van der Schans CP, v. d. M. T., de Vries G, Piers DA, Beekhuis H, Dankert-Roelse JE, et al. (1991). "Effect of positive expiratory pressure breathing in patients with cystic fibrosis." Thorax 46(4): 252-256.

van Haren EH, L. J., Festen J, Heijerman HG, Groot CA, van Herwaarden CL. (1995). "The effects of the inhaled corticosteroid budesonide on lung function and bronchial hyperresponsiveness in adult patients with cystic fibrosis." Respiratory Medicine 89(3): 209-214.

vanWinden CMQ, V. A., Hop W, Sterk PJ, Beckers S, de Jongste JC. (1998). "Effects of flutter and PEP mask physiotherapy on symptoms and lung function in children with cystic fibrosis. ." European Respiratory Journal 12(1): 143-147.

Varekojis SM, D. F., Flucke RL, Filbrun DA, Tice JS, McCoy KS, et al. (2003). "A comparison of the therapeutic effectiveness of and preference for postural drainage and percussion, intrapulmonary percussive ventilation, and high-frequency chest wall compression in hospitalized cystic fibrosis patients. ." Respiratory Care 48(1): 24-28.

Vidailhet M, D. J., Morali A, De Gasperi JP. (1987). Comparison of effectiveness and tolerance of enteric coated versus unprotected pancreatic extracts in CF patients [abstract]. 15th Annual Meeting of the European Working Group for Cystic Fibrosis, Oslo, Norway.

Vyas H, M. D., Milla PJ. (1990). "A comparison of enteric coated microspheres with enteric coated tablet pancreatic enzyme preparations in cystic fibrosis. A controlled study." European Journal of Pediatrics 149(4): 241-243.

Wambebe CO, B. E., Badru BO, Hadiza K, Momoh JA, Ekpeyong M, et al. (2001). "Efficacy of NIPRISAN in the prophylactic management of patients with sickle cell disease. ." Current Therapeutic Research 62(1): 26-34.

Wang CI, I. C., Armer C, Roldan MA, Osher AB. (1988). "Comparison of the efficacy and safety of oral ciprofloxacin with that of i.v. tobramycin plus azlocillin and/or tobramycin plus ticarcillin in patients with cystic fibrosis [abstract]. ." ExcerptaMedica, Asia Pacific Congress Series 74(R(c)): 19.

Warwick WJ, W. C. (1990). "Matched pair comparison of manual chest physical therapy (CPT) and the thairapy bronchial drainage vest (ThBVD) system [abstract]. ." Pediatric Pulmonology(Suppl 5): 177.

Warwick WJ, W. C., Hansen LG. (2004). "Comparison of expectorated sputum after manual chest physical therapy and high frequency chest compression. ." Biomedical Instrumentation Technology 38(6): 470-475.

Webber B, P. R., Hofmeyr J, Hodson M. (1985). "Evaluation of self-percussion during postural drainage using the forced expiration technique. ." Physiotherapy Theory and Practice 1(1): 42-45.

Weber AM, F. A., deGheldere B, Dufour OL, Bonin A, Roy CC. (1981). "Enteric coated microspheres of sodium bicarbonate do not enhance the effectiveness of pancrease [abstract]. ." 22nd Cystic Fibrosis Club Abstracts: 164.

Weller PH, I. D., Preece MA, Matthew DJ. (1980). "Controlled trial of intermittent aerosol therapy with sodium 2-mercaptoethane sulphonate in cystic fibrosis. ." Thorax 35(1): 42-46.

Westerman EM, B. A., Brun PP, Roldaan AC, Frijlink HW, Heijerman HG. (2008). "Aerosolization of tobramycin (TOBI) with the PARI LC plus reusable nebulizer: which compressor to use? Comparison of the CR60 to the portaneb compressor. ." Journal of Aerosol Medicine and Pulmonary Drug Delivery 21(3): 269-280.

Wiebicke W, P. A., Montgomery M, Pagtakhan R. (1990). "The effect of ipratropium bromide on lung function in patients with cystic fibrosis [Der Einfluß von Ipratropiumbromid auf die Lungenfunktion bei Patienten mit zystischer Fibrose]. ." Pneumologie 44(Suppl 1): 277-278.

Williams J, M. A., Weller PH, Fields J, Pandov H. (1990). "Two enteric coated microspheres in cystic fibrosis. ." Archives of Disease in Childhood 65(6): 594-597.

Wirth A, M. G., Braeuning Ch, Schlierf G. (1982). "Treatment of familial hyperholesterolemia with combination of bezafibrate and guar. ." Atherosclerosis 45: 291-297.

Wolfe BM, G. P. (1992). "High protein diet complements resin therapy of familial hypercholesterolemia. ." Clinical and Investigative Medicine 15(4): 349-359.

Wolter JM, B. S., Nolan PJ, McCormack JG. (1997). "Home intravenous therapy in cystic fibrosis: a prospective randomized trial examining clinical, quality of life and cost aspects. ." European Respiratory Journal 10(4): 896-900.

Young AC, W. J., Kotsimbos TC, Naughton MT. (2008). "Randomised placebo controlled trial of non-invasive ventilation for hypercapnia in cystic fibrosis. ." Thorax 63(1): 72-77.

Young G, S. F., Rojas P, Seremetis S. (2008). "Single 270 mcg/kg -dose rFVIIa versus standard 90 mcg/kg – dose rFVIIa and APCC for home treatment of joint bleeds in haemophilia patients with inhibitors: a randomized comparison. ." Haemophilia 14(2): 287-294.

Zemer D, R. M., Pras M, Modan B, Schor S, Sohar E, et al (1974). "A controlled trial of colchicine in preventing attacks of familial Mediterranean fever." New England Journal of Medicine 291(18): 932-934.

Ziebach R, P.-B. B., Bichler M, Busch A, Riethmuller J, Stern M. (2001). "Bronchodilatory effects of salbutamol, ipratropium bromide and their combination: Double-blind, placebo-controlled crossover study in cystic fibrosis. ." Pediatric Pulmonology 31(6): 431-435.

**Included in Cochrane review as a crossover study but does not have a crossover design*
