# Supplementary material for: The Use and Reporting of the Cross-Over Study Design in Clinical Trials and Systematic Reviews: A Systematic Assessment
Source: PLoS One. 2016 Jul 13;11(7):e0159014. doi: 10.1371/journal.pone.0159014 (PMC4943623; doi:10.1371/journal.pone.0159014)
Supplement: S1 Table — List of information extracted at the review level and at the study level. (DOCX) [file pone.0159014.s003.docx]

**Supplementary Table 1: Data items extracted.** List of information extracted at the review level and at the study level

| **Data extracted** | **How data was recorded** |
| --- | --- |
| **Reviews** | |
| Number of included studies | Number |
| Number of cross-over studies included | Number |
| Number of studies awaiting assessment with cross-over design | Number |
| Number of ongoing studies with cross-over design | Number |
| Number of cross-over studies excluded by design | Number |
| Methods described for the inclusion of data from crossover trials in the review (methods section)^a^ | Text description |
| Methods used for the inclusion of data from crossover trials in the review (results section)^b^ | Text description |
| **Trials^c^** | |
| Number of reviews the trial is included in | Number |
| Sample size | Number |
| Sample size calculation described? | Text description |
| Does the sample size take account of the cross-over / paired design? | Yes / No / NA (sample size calculation not performed) |
| Was a justification given for the use of a cross-over design? | Yes (provide details) / No |
| Was participant preference of treatments measured? | Yes / No |
| Design of the study | Text description |
| Washout period used or mentioned? | Yes / No / Unclear |
| Was a test for carry-over effect performed? | Yes (provide details) / No |
| Was a test for period effect performed? | Yes (provide details) / No |
| If period effect was present, were results adjusted? | Yes / No / NA (no period effect) |
| Was an appropriate statistical analysis performed?^d^ | Yes / No / Unclear |
| Details of statistical analysis performed | Text description |
| How were results reported? | Text description |
| Can reported results be included in meta-analysis?^e^ | Yes (all results) / Yes (some results) / No |
| Was an intention to treat approach used for analysis? | Yes / No / Unclear |
| Was a diagram of participant flow through the study presented? | Yes / No |
| **Trials within Reviews** | |
| Did reviewers correctly include data from the cross-over trial for the review primary outcome(s)? ^e,f^ | Yes / No / Results not included / Results included in text only |

Legend: List of information extracted at the review level and at the trial level

**Footnotes**

N= number, NA=Not applicable

1. Extracted in reviews where cross-over trials were eligible only
2. Extracted in reviews where cross-over trials were included only
3. Adapted from the list of data extracted in Mills *et al* [4]
4. The following methods of analysis were considered to be appropriate for the analysis of paired data before starting data extraction (other considered on a case by case basis): Paired t-test, Wilcoxon signed rank test, repeated measures analysis of variance (ANOVA), analysis of covariance (ANCOVA), McNemar’s test, mixed models which take account of repeated measures.
5. Data could be included in meta-analysis if results adjusted for the paired design could be extracted (e.g. mean difference and standard error (SE) of mean difference) or calculated (e.g. from individual participant data presented, from estimation of SE from exact p value reported or from correlation coefficient between treatment groups) or if data was presented by treatment period or first period only.
6. For studies included in more than one review, it was recorded whether different approaches were taken in different reviews
