# Supplementary material for: The Use and Reporting of the Cross-Over Study Design in Clinical Trials and Systematic Reviews: A Systematic Assessment
Source: PLoS One. 2016 Jul 13;11(7):e0159014. doi: 10.1371/journal.pone.0159014 (PMC4943623; doi:10.1371/journal.pone.0159014)
Supplement: S2 Table — Inclusion of cross-over trials for the primary outcomes (PO) of reviews of the Cochrane Cystic Fibrosis and Genetic Disorders Group. (DOCX) [file pone.0159014.s004.docx]

**Supplementary Table 2: Inclusion of cross-over data in Cochrane Reviews.** Inclusion of cross-over trials for the primary outcomes (PO) of reviews of the Cochrane Cystic Fibrosis and Genetic Disorders Group

| **Cross-over trial included in one review for the review primary outcome (PO) ^a^** | **190 (87%)** |
| --- | --- |
| **Trial not included in the results section of the review** | **22 (12%)** |
| Trial could not have been included in meta-analysis for the review correctly^b^ | 19 (86%) |
| *No results reported (trial is presented as an abstract only)* | *4 (21%)* |
| *No outcomes of interest (PO of the review)* | *9 (47%)* |
| *Trial authors contacted^c^* | *6 (32%)* |
| Results presented in the trial could have been included in meta-analysis correctly | 3 (14%) |
| *No outcomes of interest (PO of the review)* | *3 (100%)* |
| **Trial included narratively in the review** | **78 (41%)** |
| Trial could not have been included in meta-analysis for the review PO correctly | 61 (78%) |
| Results presented in the trial could have been included in meta-analysis correctly | 17 (22%) |
| *Individual participant data presented* | *11 (65%)* |
| *Adjusted results presented* | *4 (23%)* |
| *Data presented for first period / by treatment period* | *2 (12%)* |
| **Trial included in the review and meta-analysis as a parallel trial (incorrectly)** | **59 (31%)** |
| Trial could not have been included in meta-analysis for the review PO correctly | 48 (81%) |
| Results presented in the trial could have been included in meta-analysis correctly | 11 (19%) |
| *Individual participant data presented* | *5 (45%)* |
| *Adjusted results presented* | *6 (55%)* |
| **Trial included in the review and meta-analysis correctly** | **31 (16%)** |
| Trial could not have been included in meta-analysis for the review PO correctly | 7 (23%) |
| *Included in adjusted analyses using correlation from another study* | *1 (14%)* |
| *First period / adjusted data supplied by trialist* | *2 (29%)* |
| *Unclear where additional data has come from for adjusted analyses* | *4 (57%)* |
| Some results presented in the trial could have been included in meta-analysis correctly | 5 (16%) |
| *First period data presented* | *2 (40%)* |
| *Adjusted results available in the publication* | *3 (60%)* |
| Results presented in the trial could have been included in meta-analysis correctly | 19 (61%) |
| *First period data included:* | *11 (58%)* |
| *Individual participant data presented* | *3 (27%)* |
| *Data available for first period / by treatment period* | *8 (73%)* |
| *Adjusted analyses performed:* | *8 (42%)* |
| *Individual participant data presented* | *3 (37%)* |
| *Adjusted results presented* | *4 (50%)* |
| *First period data presented and additional adjusted data provided* | *1 (13%)* |
| **Cross-over trial included in two or more reviews^a^** | **28 (13%)** |
| **Trial not included in the results section of the reviews^d,e^** | **1 (4%)** |
| Trial could not have been included in meta-analysis for the review PO correctly | 1 (100%) |
| **Trial included narratively in the reviews** | **8 (28%)** |
| Trial could not have been included in meta-analysis for the review PO correctly | 6 (75%) |
| Could have been included in meta-analysis^d^ correctly | 2 (25%) |
| **Trial included in the reviews and meta-analyses correctly** | **1 (4%)** |
| *Adjusted results presented* | *1 (100%)* |
| **Different approaches taken in different reviews to trial inclusion** | **18 (64%)** |
| Trial could not have been included in meta-analysis for the review PO | 14 (79%) |
| *Analysed as a parallel trial / not included and authors contacted^c^* | *1 (7%)* |
| *First period data included or adjusted analyses performed with extra data provided by trialists* | *2 (14%)* |
| *Included narratively / not included and authors contacted^c^* | *4 (29%)* |
| *Included narratively / analysed as a parallel trial* | *5 (36%)* |
| *Included narratively / adjusted analyses performed with extra data provided by trialists* | *1 (7%)* |
| *Analysed as parallel / adjusted analyses performed with extra data provided by trialists* | *1 (7%)* |
| Some results presented in the trial could be included in meta-analysis | 1 (4%) |
| *Included narratively / adjusted analyses from individual participant data* | *1 (100%)* |
| Results presented in the trial could have been included in meta-analysis | 3 (17%) |
| *Included narratively / included in adjusted analyses* | *1 (33%)* |
| *First period data included / not included and authors contacted^c^* | *1 (33%)* |
| *First period data included or adjusted analyses performed with extra data provided by trialists* | *1 (33%)* |

Legend: Inclusion of cross-over trials for the primary outcomes (PO) of reviews of the Cochrane Cystic Fibrosis and Genetic Disorders Group.

**Footnotes**

1. Total of 218 cross-over trials
2. We define ‘correctly’ as an analysis method which accounts for the cross-over design of the trial (use of adjusted analyses or inclusion of first period data only). We define ‘incorrectly’ as not accounting for the cross-over design (e.g. analysing as a parallel trial).
3. In one review, cross-over trials were included in meta-analysis only if a clear and “sufficient” washout period was described, therefore for the majority of cross-over trials included in the review, trial authors were contacted for further details about washout periods.
4. No primary outcomes of one of the reviews were reported in the trial.
5. One of the reviews includes first period data only and no first period data was available for the trial.
